# Supplementary material for: Improving the generalizability of protein-ligand binding predictions with AI-Bind
Source: Nat Commun. 2023 Apr 8;14:1989. doi: 10.1038/s41467-023-37572-z (PMC10082765; doi:10.1038/s41467-023-37572-z)
Supplement: Supplementary file 1 — Supplementary Information [file 41467_2023_37572_MOESM1_ESM.pdf]

Improving the generalizability of protein-ligand binding predictions with AI-Bind

Ayan Chatterjee[1], Robin Walters[2], Zohair Shafi[2], Omair Shafi Ahmed [2], Michael Sebek[1,3], Deisy Gysi[1,3,4], Rose Yu[5], Tina Eliassi-Rad[1,2,6,7], Albert-László Barabási[1,3,8], Giulia Menichetti[1,3,9,*]

[1]Network Science Institute, Northeastern University, Boston, USA
[2]Khoury College of Computer Sciences, Northeastern University, Boston, USA
[3]Department of Physics, Northeastern University, Boston, USA
[4]Department of Medicine, Brigham and Women's Hospital, Harvard Medical School, Boston, USA
[5]Department of Computer Science and Engineering, University of California, San Diego, USA
[6]Santa Fe Institute, Santa Fe, NM, USA
[7]The Institute for Experiential AI, Northeastern University, Boston, MA, USA
[8]Department of Network and Data Science, Central European University, Budapest, Hungary
[9]Channing Division of Network Medicine, Department of Medicine, Brigham and Women's Hospital, Harvard Medical School, Boston, USA
[*]Corresponding author e-mail: giulia.menichetti@channing.harvard.edu

SUPPLEMENTARY INFORMATION

Table of Contents

Supplementary Note 1: Emergence of topological shortcuts **4**

Supplementary Note 2: Naturally occurring ligands **9**

Supplementary Note 3: DeepPurpose false negative predictions due to annotation imbalance **9**

Supplementary Note 4: Databases **10**

DrugBank 10

Drug Target Commons 10

BindingDB 10

Natural Compounds in Food Database 10

Supplementary Note 5: 7-hop threshold for network-derived negatives **11**

Supplementary Note 6: Novel deep learning models **12**

VecNet 13

Siamese model 14

VAENet 14

Supplementary Note 7: Additional deep learning model results **15**

Supplementary Note 8: Comparison with MolTrans **16**

Supplementary Note 9: Interpretability of AI-Bind: Identifying active binding sites **16**

Supplementary Note 10: Validation using gene phylogeny and bias in false predictions **18**

Supplementary Note 11: Optimal representation of protein and ligand molecules **18**

Supplementary Note 12: Random Negative Sampling **20**

Supplementary Note 13: Gold standard validation of binding probability profile **21**

Supplementary References **22**

Supplementary Note 1: Emergence of topological shortcuts

Decision rules learned by many machine learning (ML) models tend to perform well on benchmark datasets, but fail to generalize well when given never-before-seen data. Instead of learning generalizable patterns from features observed during training, these models leverage shortcuts in the data to maximize transductive performance, i.e., the performance on seen data [1]. In this section, we investigate how the properties of the network data used in training can drive ML models to learn topological shortcuts, rather than taking into account node features that would allow better generalizability to unseen data. We assess the emergence of topological shortcuts by null models (configuration model) achieving good transductive test performance.

BindingDB data observations

First, we investigated the statistical properties of the training database used by DeepPurpose [2], a modeling pipeline offering state-of-the-art neural architectures to predict protein-ligand binding. The training data is based on all the records in BindingDB [3] characterized by the kinetic disassociation constant $\mathrm{K_d}$. The distribution of the number of annotations per protein $P(k_p)$ is well fitted by a power law distribution using [4]

$$P(k_p) \sim k^{-\gamma_p}, \tag{1}$$

with $\gamma_p = 2.84$, $k_p^{min} = 1$, and $k_p^{max} = 1{,}426$ (Figure 1a in the main text). We make similar observations for the ligands, with $\gamma_l = 2.94$, $k_l^{min} = 1$, and $k_l^{max} = 1{,}161$.

From the original annotations in BindingDB, a binary classification dataset is derived using a threshold of 30 nM for $\mathrm{K_d}$ [2]. Protein-ligand pairs with $\mathrm{K_d} < 30\ nM$ are binding or positive pairs, and everything else is labeled as non-binding or negative. Overall, we observe that 16% of the records are labeled as positive, a characteristic of the database that we summarize as $p_{bind} = 0.16$, the probability to observe a binding annotation independently from the identity of the protein and the ligand. Furthermore, We find that it is easier to capture the properties of the $\mathrm{K_d}$ distribution in BindingDB by modeling it as a log-normal (Supplementary Figure 1a).

Additionally, we find that the number of annotations k and the average disassociation constant $\langle \mathrm{K_d} \rangle$ over the links associated with the nodes of degree k are not independent but show a negative rank correlation. In particular, for proteins we find $r_{Spearman}(k_p, \langle \mathrm{K_d} \rangle) \approx -0.47$ (Supplementary Figure 1b), and for ligands $r_{Spearman}(k_l, \langle \mathrm{K_d} \rangle) \approx -0.29$. Alongside this negative correlation, we observe a higher variance in both the linear and logarithmic K_d values across

links originating from low-degree nodes compared to higher-degree nodes (Supplementary Figures 1d and 1f). The relation between degrees and kinetic constants makes the link prediction task easier for the hubs compared to the low-degree nodes, leveraging only degree information. Since most links in the network are associated with the hubs, the configuration model is able to achieve excellent transductive test performance.

Toy model set-up

In order to test our hypotheses regarding the creation of topological shortcuts, we simulate synthetic network data that we call *toy models* (Supplementary Figure 2). We create a duplex of unipartite networks with features inspired by the protein sample captured in BindingDB, as similar considerations extend to bipartite networks. Specifically, we vary the degree distribution $P(k)$ and $r_{Spearman}(k, \langle \mathrm{K_d} \rangle)$ to explore when the output of the duplex configuration model $\{p_{ij}^{conditional}\}$ (Equation (7) in the main text) becomes highly variable and thus informative, creating the potential for topological shortcuts (Figure 3a and Methods). In other words, the closer $\{p_{ij}^{conditional}\}$ gets to a Naive Bayes classifier, the less attractive it will be for machine learning models learning a classification task as the predictions would provide information with no discrimination power.

We generate random duplexes of unipartite networks with Poisson or power-law degree distributions and different correlations $r_{Spearman}(k, \langle \mathrm{K_d} \rangle)$ according to four different specifications:

- Poisson degree distribution and $r_{Spearman}(k, \langle \mathrm{K_d} \rangle) \approx -0.47$;
- Poisson degree distribution and $r_{Spearman}(k, \langle \mathrm{K_d} \rangle) \approx 0$;
- Power law degree distribution and $r_{Spearman}(k, \langle \mathrm{K_d} \rangle) \approx -0.47$;
- Power law degree distribution and $r_{Spearman}(k, \langle \mathrm{K_d} \rangle) \approx 0$.

We generate random unipartite toy networks inspired by the topological and kinetic features of the protein training data used in DeepPurpose. We fix the size of the network to $N = 1,507$ and we create randomized networks using the same degree sequence as in BindingDB. For the Poisson case, the link density is constrained by the average number of annotations in the original network. The weight ${\mathrm{K_d}}^{(i,j)}$ assigned to edge (i, j) represents a kinetic constant, and it is derived as the geometric mean of the contribution ${\mathrm{K_d}}^{i}$ from node i and the contribution ${\mathrm{K_d}}^{j}$ from node j, namely,

$$K_d^{(i,j)} = \sqrt{K_d^i K_d^j}. \tag{2}$$

We explore multiple scenarios to validate our hypothesis on the emergence of topological shortcuts: in presence of an anti-correlated relation between k and $\langle \mathrm{K_d} \rangle$ as observed in BindingDB, affecting both average values and degree-dependent fluctuations in $\mathrm{K_d}$, only power-law networks will lead to topological shortcuts. To simplify the modeling of different correlation structures, we use the log-space of kinetic constants, and explore four different sampling strategies: (i) sampling without any variance in the log $\mathrm{K_d}^i$ values contributed by node i to its links where the sampled log $\mathrm{K_d}^i$ values are selfsame for all nodes i with a given degree k, (ii) sampling without any variance in the log $\mathrm{K_d}^i$ values contributed by node i to its links in presence of node-level fluctuations for the sampled log $\mathrm{K_d}^i$, (iii) sampling with variance in the log $\mathrm{K_d}^i$ values, the variance being independent of the degree k of node i and equal to logarithmic variance of BindingDB annotations, (iv) sampling with variance in the log $\mathrm{K_d}^i$ values, with the variance decreasing as a function of k, as observed in the BindingDB data.

According to the sampling strategy, each node contributes to Supplementary Equation (2) for all the associated edges with a different extent of variability. In particular, in scenarios (i) and (ii) each node is assigned to a single log $\mathrm{K_d}^i$ for all its edges, sampled according to its degree k. Additionally, in (ii), for a fixed degree k, log $\mathrm{K_d}^i$ is sampled from a normal distribution with mean $m = \mu + r_{Spearman} * \sigma * \Phi^{-1}(k)$ and standard deviation $s = \sqrt{}((1 - r^2_{Spearman}) * \sigma^2)$, where μ and σ are the mean and standard deviation of the log $\mathrm{K_d}$ values in BindingDB, and Φ^{-1} is the inverse cumulative distribution function of a standard normal distribution. In (iii) we follow a similar approach to (ii), but instead of sampling a single value, we assign to each node a sample of 5,000 i.i.d. log $\mathrm{K_d}$ instances. Thereafter, when assigning log $\mathrm{K_d}^i$ to each link, we sample uniformly from the generated list of values. In scenario (iv), to generate a final network with edge-level $\mathrm{K_d}$ fluctuations compatible with real data observations in Supplementary Figures 1c-f, we leverage as input the observed $\mathrm{K_d}$ fluctuations at node level per degree k. Indeed, this strategy is a good heuristic to generate networks compatible with BindingDB data, given the subsequent error propagation due to the edge-level $\mathrm{K_d}$ computation using the geometric mean. Specifically, we first calculate the mean $\langle \log \mathrm{K_d} \rangle$ and the standard deviation $\sigma_{\log K_d}$ for all unique k values at the node level. Then, for each link associated with node i we sample a log $\mathrm{K_d}^i$ value from a normal distribution with mean and standard deviation equal to the parameters corresponding to the degree of node i.

We select as threshold for $\mathrm{K_d}^{(i,j)}$ the value for which 16% of the annotations become positive (binding), enforcing the constraint on the observed $p_{bind} = 0.16$. Based on this threshold, we generate the duplex layers with positive and negative edges and calculate the multilink degree sequences, input to the configuration model (see Methods). For the uncorrelated version, we

fix the topology while shuffling the ${K_d}^{(i,j)}$ values at random, which removes any correlation between k and $\langle K_d \rangle$.

Mathematical formalism for the uncorrelated scenario

When $\langle K_d \rangle$ and k are independent, we can analytically derive the statistical behavior of positive degree k^+, negative degree k^-, and degree ratio ρ (Equation (1) in the main text). For each node, the probability of observing k^+ positive annotations out of k links is binomial

$$P(k^+|k) = \binom{k}{k^+} p_{bind}^{k^+}(1-p_{bind})^{(k-k^+)}, \tag{3}$$

where p_{bind} encodes the percentage of positive records observed in the database.

The distribution of positive annotations k^+ for the whole database is then a compound distribution

$$P(k^+) = \int P(k)P(k^+|k)dk, \tag{4}$$

where $P(k)$ is the candidate probability distribution for the number of annotations k.

From the laws of total expectation and total variance we derive

$$\begin{aligned} \langle k^+ \rangle &= p_{bind}\langle k \rangle, & (5)\\ \sigma^2(k^+) &= p_{bind}(1-p_{bind})\langle k \rangle + p_{bind}^2(\langle k^2 \rangle - \langle k \rangle^2), & (6) \end{aligned}$$

where similar equations hold for k^-, with $(1-p_{bind})$ replacing p_{bind}. When $P(k)$ is fat-tailed, $(\langle k^2 \rangle - \langle k \rangle^2)$ becomes dominant, and the random variable $k^+ \approx p_{bind}k$. This formulation suggests that, even in the presence of fat-tailed $P(k)$, the lack of correlations between $\langle K_d \rangle$ and k would determine a distribution of degree ratio ρ well represented by the average

$$\langle \rho \rangle = p_{bind}, \tag{7}$$

with noise determined by p_{bind} and $P(k)$. As the duplex configuration model constrains the degree ratio sequence $\{\rho_i\}$, the variability of $\{p_{ij}^{conditional}\}$ drops significantly in absence of correlation between $\langle K_d \rangle$ and k, bringing the model closer to a Naive Bayes classifier (Supplementary Figure 3).

We can clearly derive the behavior of $p_{ij}^{conditional}$ in the case of *uncorrelated networks*, i.e., networks with no degree correlation and an upper bound for the maximum degree equal to $\sqrt{\langle k \rangle N}$, where N is the size of the unipartite network (Advanced Topics 7.B in [5]). In this

scenario the Lagrangian multipliers satisfy:

$$e^{-\lambda_i^{(1,0)}} = \frac{k_i^+}{\sqrt{\langle k^+\rangle N}} \approx \frac{p_{bind}k_i}{\sqrt{p_{bind}\langle k\rangle N}}, \quad (8)$$

$$e^{-\lambda_i^{(0,1)}} = \frac{k_i^-}{\sqrt{\langle k^-\rangle N}} \approx \frac{(1-p_{bind})k_i}{\sqrt{(1-p_{bind})\langle k\rangle N}}, \quad (9)$$

$$p_{ij}^{(1,0)} = e^{-(\lambda_i^{(1,0)}+\lambda_j^{(1,0)})}, \quad (10)$$

$$p_{ij}^{(0,1)} = e^{-(\lambda_i^{(0,1)}+\lambda_j^{(0,1)})}. \quad (11)$$

It follows that Equation (7) in the main text for $p_{ij}^{conditional}$ in the transductive test becomes independent from the identity of node i and j, as the product k_ik_j simplifies, leading to $p_{ij}^{conditional} \approx p_{bind}$.

Observations

The major driving factor in the emergence of topological shortcuts is the relation between k and $\langle \mathrm{K_d}\rangle$. The monotonicity of the relation between k and $\langle \mathrm{K_d}\rangle$ helps the configuration model to predict the link probabilities using the degree sequence as the $\mathrm{K_d}$ values associated with the links are directly associated with the link labels after thresholding. In scenarios (i) "strictly no fluctuation" and (ii) "no fluctuation", when k and $\langle \mathrm{K_d}\rangle$ values are anti-correlated and $\langle \mathrm{K_d}\rangle$ values have no or negligible fluctuations for a fixed k, degree becomes a strong predictor of $\mathrm{K_d}^{(i,j)}$ and subsequently the link types. Hence we observe excellent transductive test performance of the configuration model, for any topology (Supplementary Table 1 and Supplementary Figure 3). Similar performance of scenarios (i) and (ii) suggests that the transductive performance is unaffected by node level fluctuations in $\mathrm{K_d}$ and that tuning the link level fluctuations unveils the mechanism behind topological shortcuts, bolstering the construction of our toy model.

In scenario (iii) "constant fluctuations", when we introduce variance over the $\langle \mathrm{K_d}\rangle$ values, the monotonic relation between k and the link types is disrupted. Hence it is difficult for the configuration model to predict the link type only using the degree information. This observation is consistent for networks with both power law and Poisson degree distributions. Yet, we observe that the variance of $\mathrm{K_d}$ is not uniform for different k values in BindingDB. The hubs encounter less variance in $\mathrm{K_d}$ compared to the low-degree nodes, which we simulate in scenario (iv) "varying fluctuations". Thus, the configuration model is able to predict the link types associated with the hubs. Since these hubs are associated with the majority of the links in the protein-ligand interaction network and these links constitute most of the samples in both train and test datasets under a random edge split, making correct predictions using only the degree information of

the hubs helps the configuration model achieve commendable transductive test performance. The performance drops for networks with Poisson degree distributions, where hubs are absent, despite enforcing the same type of correlation structure. When we remove the anti-correlation between k and $\langle \mathrm{K_d} \rangle$, irrespective of the variance of $\langle \mathrm{K_d} \rangle$ values, the configuration model fails to predict the link types using only the degree information. In this scenario, the configuration model performs similar to a Naive Bayes classifier. Given the observed correlation structure in BindingDB (real-world scenario), which affects both expected values and fluctuations of the kinetic constants, topological shortcuts emerge in presence of power law.

Supplementary Note 2: Naturally occurring ligands

We extend the drug repurposing task to additional ligands which are not necessarily considered drugs but may nonetheless bind to protein targets. Specifically, we look into the *Natural Compounds in Food Database (NCFD)* (see Supplementary Note II), which contains food-borne natural compounds, some of which are potential protein binders. Although these ligands have known chemical structures, they lack adequate binding annotations for training ML models. Binding predictions for these ligands largely depend on comparing their chemical features to other ligands, for which more binding data is available. Supplementary Figure 4 shows that the naturally occurring compounds in NCFD are larger in size and are more diverse in terms of atomic constituents compared to the drug molecules in DrugBank. This suggests that the binding prediction task on these natural compounds is challenging, which we tackle by maximizing the amount of training data for these natural compounds, and pre-training the chemical embeddings on large chemical libraries.

Supplementary Note 3: DeepPurpose false negative predictions due to annotation imbalance

A false negative prediction corresponds to a low binding probability output by the ML model for a protein-ligand pair which does, in fact, bind. In Supplementary Figure 5a and Supplementary Figure 5b, we observe that DeepPurpose produces false negative predictions more often for ligands and proteins with low degree ratios. We notice the opposite for the false positives; nodes with high degree ratios contribute more to the false positive predictions in DeepPurpose predictions (see Figure 2c in the main text).

Supplementary Note 4: Databases

AI-Bind combines data from four databases: DrugBank, Drug Target Commons (DTC), BindingDB, and Natural Compounds in Food Database (NCFD).

DrugBank

DrugBank [6] consists of 7,307 drugs and 4,762 protein targets, which form 25,373 drug-target binding pairs. 167 of these drugs are found in NCFD, and we classify them as naturally occurring and food-borne. We consider all reported protein-ligand pairs from DrugBank as positive samples in our dataset, except 53 pairs which have kinetic constants $\geq 10^6\ nM$ in BindingDB. The protein sequences included in DrugBank are derived from a wide variety of organisms, including human and different viruses.

We observe that the annotation distribution of the proteins and the ligands in DrugBank is fat-tailed (Supplementary Figure 6a). This observation is similar to the annotation distributions in BindingDB. The fat-tailed nature of the degree distribution in the binding datasets is a result of the experimentation associated with studying protein-ligand binding. Some proteins and ligands are indeed studied more than others, and hence appear as hubs in such datasets.

Drug Target Commons

We use Drug Target Commons (DTC) [7] for obtaining binding information related to the natural compounds in NCFD. The intersection of NCFD and DTC contains 1,820 natural ligands and 466 associated proteins.

BindingDB

BindingDB [3] consists of protein-ligand pairs along with associated kinetic constants and physical conditions related to the reactions such as pH and temperature. We use BindingDB to extend the number of binding pairs in our training data, filter out the non-binding ones from DrugBank, and obtain absolute negative samples.

Natural Compounds in Food Database

Multiple existing databases contain information about the compounds present in different food items. As a part of the Foodome project at Center for Complex Network Research (CCNR), we curated external databases like FooDB [8], Dictionary of Food Compounds (DFC) [9], and KNApSAcK [10] to gather information about the compounds in food. Metabolomic experiments

were performed to further enrich the database. NCFD contains 20,700 compounds found in different food items, among which ≈ 19,000 contain isomeric SMILES [11], a plain-text encoding of the chemical structures of each molecule[1]. AI-Bind uses SMILES as input to its ML models for learning useful chemical embeddings.

Supplementary Figure 6b shows the detailed breakdown of the protein-ligand binding pairs obtained from different databases.

Supplementary Note 5: 7-hop threshold for network-derived negatives

We use shortest path distances to generate negative samples. We consider the node pairs which have shortest path distance ≥ 7 in the network as non-binding. We derive this 7-hop threshold based on two observations. First, 7 hops is the minimum shortest path distance at which the average kinetic constant value (K_{i}) is above the non-binding threshold of 10^6 nM (Figure 5d in the main text). We test our hypothesis with K_{i} values since

we lack sufficient data points for other kinetic constants (K_{d}, IC_{50}, EC_{50}) at the intersection of the BindingDB data and the protein-ligand bipartite network used in AI-Bind training. Second, 7 hops is small enough that the negative samples for a given node are not easily distinguishable from positive samples, making the learning task more complex, which helps to defeat shortcut learning in ML models. The latter observation is based on EigenSpokes [12] analysis, a network-based dimensionality reduction procedure inspired by Principal Component Analysis (PCA). Let A be the square adjacency matrix of the protein-ligand network. Since A is real symmetric, it is orthogonally diagonalizable. Let $e_1, \ldots, e_n$ be the eigenvectors of A sorted by eigenvalue magnitude $|\lambda_1| \geq |\lambda_2| \geq \ldots \geq |\lambda_n|$. Given a node i, we write the row a_i of A in terms of the eigenbasis $a_i = u_{i1}e_1 + \ldots + u_{in}e_n$. Truncating after the first 5 eigenvectors (with highest eigenvalue magnitudes) gives a low-dimensional embedding $\bar{u}_i = (u_{i1}, u_{i2}, u_{i3}, u_{i4}, u_{i5})$ of each node. The choice of 5 dimensions gives a useful low-dimensional embedding, while still capturing the most significant degrees of variation.

Now, consider a fixed protein i. Then ligands $\{j_1, j_2, ...\}$ which bind to i (1-hop) have high magnitude and variance in this 5-dimensional space. On the other hand, ligands $\{k_1, k_2, ...\}$ that are at a distance of 13 hops from i have $\bar{u}_k$ very close to the origin (Supplementary Table 2). When 13 hops is chosen as the threshold for negative samples, it would thus be trivial for ML models to distinguish nodes $\{j_1, j_2, ...\}$ 1 hop away apart from nodes $\{k_1, k_2, ...\}$ 13 hops away, resulting in shortcut learning. Indeed, the same low-degree ligands on the periphery of

[1]NCFD data was accessed on 7.14.2021. As this database undergoes constant change, we have included a description of the dataset at the time of download in the SI.

the network would become negative samples for all the proteins.

We observe a similar behavior for 11-hop and 9-hop thresholds. However, at 7 hops, we see significantly higher magnitude and variance in $\bar{u}_k$, indicating more diverse negative samples for each protein. In Supplementary Figure 7a, we visualize (u_3, u_4) for ligands, colored based on the hop-distances from the example protein BPT4. We see that at shortest path distances ≥ 7, most nodes are very close to the origin. In Supplementary Figure 7b, we show the mean of all $\|\bar{u}_j\|$ values averaged over all pairs (i, j) of a given path length. Similarly to what we observed for BPT4, we observe a significant fall-off in magnitude as the shortest path length increases.

Supplementary Note 6: Novel deep learning models

We observe that neural networks exploit the topology of the protein-ligand bipartite network used in training to achieve good performance, and lack node-level generalizability when trained in an end-to-end fashion. AI-Bind circumvents these issues by training its ML models in two phases. First, AI-Bind learns the node features using unsupervised pre-training, and then it separately trains its classifiers in a supervised manner to predict binding. To show that AI-Bind is not specific to a certain neural network architecture, we experiment with 3 two-phase networks: VecNet, Siamese model, and VAENet. AI-Bind first trains a neural network in an unsupervised manner to embed the nodes into a low-dimensional latent space, learning generalizable node representations based on the node features alone (chemical structures of ligands and amino acid sequences of proteins). For example, one of the AI-Bind architectures, VecNet, uses unsupervised node representations from Mol2vec [13] and ProtVec [14], which are trained separately from each other and from the protein-ligand bipartite network used in training. Mol2vec and ProtVec are both based on Word2Vec [15], and are designed to create low-dimensional vector representations which retain contextual information for "words" in "sentences", where the "sentences" are formed by molecular sequence descriptions such as Morgan fingerprints [16] or protein sequences. In the second phase, these node representations are passed as input to a binding prediction network, which is trained in a supervised manner. In AI-Bind's VecNet, the binding prediction network uses fully-connected layers and ReLU non-linearities.

The Siamese model uses triplet similarity to find a representation for the node (protein and ligand) features based on their common bindings. The embeddings are then used as inputs to a multilayer perceptron, which learns bindings in a separate supervised training. The last of AI-Bind's three models, VAENet, uses a Variational Auto-Encoder [17] in order to learn unsupervised ligand representations.

VecNet

We use the pre-trained Mol2vec [13] and ProtVec [14] models for node representations. The pre-trained Mol2vec and ProtVec models create 300 and 100-dimensional embeddings for ligands and proteins, respectively. They are based on Word2Vec [15], and treat the Morgan fingerprints [16] and amino acid sequences as sentences in which substructure fingerprints (fragments) and trigrams are the words, respectively. They are trained in an unsupervised manner to create the representations independently of the binding information. Namely, they are trained to predict which words appear near each other in sentences.

Given a fingerprint x^0 and an amino acid sequence x^1, we encode them using Mol2vec and ProtVec, and then pass them through a simple decoder. We experimented with different neural network architectures with differing number of layers (up to 6 dense layers) and number of neurons per layer (selected from powers of 2 starting at 128 to 2048) and picked one that performed best in inductive tests. This architecture is shown in Supplementary Figure 8a.

More formally, VecNet computes :

$$\bar{x}^0 = \texttt{mol2vec}(x^0) \in \mathbb{R}^{300}, \qquad \bar{x}^1 = \texttt{protvec}(x^1) \in \mathbb{R}^{100}$$

$$\tilde{x}^0 = \texttt{ReLU}(W^0 \bar{x}^0) \in \mathbb{R}^{2048}, \qquad \tilde{x}^1 = \texttt{ReLU}(W^1 \bar{x}^1) \in \mathbb{R}^{2048}$$

$$\begin{aligned} h^0 &= \texttt{Concatenate}(\tilde{x}^0, \tilde{x}^1) \in \mathbb{R}^{4096} \\ h^1 &= \texttt{ReLU}(W^2 h^0) \in \mathbb{R}^{512} \\ h^2 &= \texttt{ReLU}(W^3 h^1) \in \mathbb{R}^{512} \\ \hat{y} &= \sigma(W^4 h^2) \in [0, 1] \end{aligned}$$

where σ is the sigmoid function and $\sigma(x) = \frac{1}{1+e^{-x}}$.

Prior use of Mol2Vec and ProtVec in binding prediction

Mol2Vec has previously been used for binding prediction, but only for pre-specified proteins [13], where the ML model is trained on one protein at a time. No information is encoded regarding the protein except for its binding scores with other chemicals in the training data. In contrast, AI-Bind's VecNet attempts to generalize for different proteins, which we encode using ProtVec. Jaeger et al. [13] also propose PCM2vec, in which they predict properties of proteins by concatenating Mol2Vec and ProtVec vectors for the same protein read in as a molecule and amino acid sequence, respectively. However, they do not attempt to combine these vectors for

different inputs corresponding to a protein-ligand pair.

Siamese model

The Siamese model uses one-shot learning to embed proteins and ligands into the same latent space [18]. For a given node, the Siamese model minimizes the Euclidean distances of that node from the nodes which bind to it, while maximizing the distances to the nodes which do not. This process is executed in triplets of the forms ⟨protein, non-binding ligand, binding ligand⟩. For the first kind, AI-Bind trains the network to maximize the Euclidean distance between the protein target and the non-binding ligand, while minimizing the distance of the target from the binding ligand (Supplementary Figure 9a). AI-Bind uses these embeddings, generated by the Siamese architecture, to train a separate model for the downstream classification task of predicting binding. We studied the inductive test performance by changing the number of convolutional layers and the number of embedding dimensions. The final Siamese model consists of 4 convolutional layers and creates 128-dimensional output vectors. The classification network concatenates the embeddings for a protein and a ligand, and then passes it through two fully connected layers, similar to VecNet, to predict the binding probabilities (Supplementary Figure 8c).

VAENet

VAENet uses a Variational Auto-Encoder [17], an unsupervised learning technique, to embed ligands onto a latent space. Morgan fingerprints are directly fed into a convolutional neural network. The auto-encoder minimizes the loss of structural information while reconstructing the molecule back from the latent representation (Supplementary Figure 9b). We generate 300-dimensional ligand embeddings using the auto-encoder, which is consistent with the dimensionality of the Mol2vec embeddings used in VecNet. The variational nature of this 300-dimensional space allows it to be continuous, allowing for better generalizability. We achieve this generalizability by using the re-parameterization trick from [19] to sample from the latent space, instead of directly connecting the latent space to the decoder. Having a generalizable continuous space allows us to map novel ligands into the latent space.

The downstream classification task is achieved by training a fully connected neural network on the concatenated embeddings generated from the Variational Auto-Encoder and ProtVec. The non-end-to-end nature of this architecture ensures that the learned molecular features are independent from the classification task, which has a tendency to exploit shortcuts related to the topology of the protein-ligand interaction network. We observe lower performance for

VAENet compared to VecNet (Supplementary Table 3) mainly for two reasons: (i) VAE has a smaller training dataset of ~ 9 million chemicals from ZINC, whereas Mol2vec uses 19.9 million chemicals in training. Thus, Mol2vec is better at generalizing to unknown ligand structures. (ii) VAE uses an auto-encoder to embed the ligand molecules, which is a dimensionality reduction approach. Mol2vec uses skipgrams to embed the molecular structures, which is better at capturing contextual information for different fragments in the molecule and provides a more intelligible representation of the ligand structures for the downstream classification task.

Supplementary Note 7: Additional deep learning model results

Supplementary Table 3 contains the performances of AI-Bind's novel deep learning architectures, a DeepPurpose baseline (Transformer-CNN), and the duplex network configuration model on the network-derived dataset. We also report the performances for models trained with randomized node features. This removes structural information about the proteins and ligands, helping us understand whether the deep learning models leverage structure to learn binding or take topological shortcuts. We observe that DeepPurpose's performance does not change if the inputs are randomly shuffled, which suggests that DeepPurpose learns the topology of the protein-ligand interaction network instead of the node features (Table 3 in the main text).

In AI-Bind, network-derived negatives and unsupervised pre-training allow the deep learning models to learn binding patterns using the chemical structures instead of the topology of the protein-ligand interaction network. Thus, we observe diminished performance while using random features to make predictions for unseen nodes (inductive test). In this case, network-derived negatives remove the annotation imbalance from the training data and prohibit the ML models from taking topological shortcuts.

Supplementary Figure 10 shows the training curves averaged over 5 data splits ($85 : 15$ split to create train and validation-test datasets) for AI-Bind's three novel models. We set the stopping criterion for training to maximize the inductive test performance (AUPRC) on the validation set. Supplementary Figure 11 shows the F1-scores for the trained VecNet model relative to the classification threshold. We obtain the optimal threshold from this curve, which corresponds to the highest F1-score. This optimal threshold is used to obtain the binary labels from the predicted continuous outputs of the AI-Bind architectures. For AI-Bind's VecNet, we obtain an optimal threshold of 0.09 ($\pm$ 0.015) in the inductive test scenario. We observe a low optimal threshold as AI-Bind's VecNet predicts high binding probability (p_{ij}^{VecNet}) for a few protein-ligand pairs, but we have roughly the same number of positive and negative samples in

the test data. We recommend to use p_{ij}^{VecNet} values to select the top-N predictions, rather than using this optimal threshold to derive the binary labels for novel protein-ligand pairs absent in AI-Bind test data.

Supplementary Note 8: Comparison with MolTrans

We compare the performance of AI-Bind with the Molecular Interaction Transformer (MolTrans) [20], a state-of-the-art protein-ligand binding prediction model which uses a combination of sub-structural pattern mining algorithm, interaction modeling module, and an augmented transformer encoder to better learn the molecular structures. Innovative representation of the molecules improves the transductive test performance upon DeepPurpose. MolTrans achieves transductive AUROC of 0.950 ± 0.041 and AUPRC of 0.888 ± 0.087 on the BindingDB data, while DeepPurpose achieves transductive AUROC of 0.818 ± 0.004 and AUPRC of 0.482 ± 0.004. However, MolTrans performs poorly in inductive tests, i.e., while predicting over novel proteins and ligands. We observe that AI-Bind's VecNet performs better than both DeepPurpose and MolTrans in transductive, semi-inductive, and inductive tests. VecNet's improved inductive performance validates that unsupervised pre-training improves the generalizability of the protein-ligand binding models. The results are summarized in Supplementary Table 4.

Supplementary Note 9: Interpretability of AI-Bind: Identifying active binding sites

AI-Bind may be used to find active binding sites on the amino acid sequence. We plan to leverage this information to define an optimal search grid for docking simulations. Specifically, we use AI-Bind to identify which trigrams in the amino acid sequence are most significant in predicting binding, thus indicating potential binding locations. This is achieved via an ablation study [21], where each trigram in the input amino acid sequence is mutated, that is, replaced with *xxx*, which maps to the *unknown* vector $\langle unk \rangle$ in the ProtVec model. The *unknown* vector is a learned 100-dimensional vector to which all out-of-vocabulary entries, i.e., amino acid trigrams not present in the ProtVec training corpus, are mapped. This *unknown* vector is set to the mean of all the other learned vectors. We predict the probable binding locations by mutating each trigram in the amino acid sequence one at a time and observing the fluctuations in the AI-Bind predictions (Supplementary Figure 12a). We then smooth the fluctuations using a moving average with a window size of 10 (to eliminate the auxiliary valleys) and obtain a *binding probability profile*.

The suggested binding sites correspond to the amino acid trigrams in the valleys of the binding probability profile. We validate that ligands bind at these valleys by visualizing the docking outputs (see Results) using PyMOL [22] and identifying the region around the ligand with a radius of 5Å, corresponding to the active binding sites (Figure 6c). These regions enclose the amino acid residues which form different bonds with the ligand molecule. Bond distances are measured between the centers of two atoms. Length of hydrogen bonds are typically between 2.3 and 3.9Å [23, 24]. London dispersion forces or Van der Waals interaction between non-polar chains have bond length between 3.8 to 4.2Å [25]. Thus, selecting a sphere with a radius of 5Å around the ligand encloses all possible bonds between the ligand and the protein. We identify the amino acid residues inside this sphere, map them to the regions on the amino acid sequence and compare the results with the valleys in the binding probability profile.

To test this method in a specific case, we identify the active binding sites on the human protein Trim59. For Pipecuronium, Buprenorphine and Voclosporin, three ligands binding to Trim59 at three different pockets, we study the valleys in the binding probability profile which predict binding locations on the amino acid sequence (Figure 6c). More generally, considering a broad range of ligands, we predicted a total of four active binding sites on the protein Trim59, three of which have been validated in the docking simulations (Supplementary Figure 12b). We group the ligands binding to Trim59 according to the different binding sites (Supplementary Figure 13). From our analysis, a possible fourth active binding site emerges, based on a valley in the binding probability profile, but not associated with any ligand tested in the docking simulations. The shape of the binding probability profiles remains the same across different ligands, but the drop from the original VecNet prediction (depth of a valley or Δp_{ij}^{VecNet}) fluctuates for different ligands (Supplementary Figure 13). We observe a moderate positive correlation between the depth of the valleys and binding affinities ($r_{Spearman}(\Delta p_{ij}^{VecNet}, \Delta G) = 0.13$). This indicates the depth of the valleys could be indicative of the binding strength and help in identifying the exact binding site on the protein.

Furthermore, we performed unsupervised hierarchical clustering on the binding probability profiles for different ligands of Trim59. By clustering first the ligand structures using Tanimoto similarity [26], we find that the ligands binding to Trim59 are diverse in structure, irrespective of the pockets they bind to (Supplementary Figure 14a). Thus, we cannot identify the specific binding pocket using only the ligand structure. When we compare this result to the clustering emerging from the binding probability profiles, we observe a grouping more correlated with the pocket labeling (Supplementary Figure 14b).

Supplementary Note 10: Validation using gene phylogeny and bias in false predictions

As additional validation, we investigate if AI-Bind's VecNet predictions are biased towards certain protein structures. The inductive test sets contain a total of 4,583 proteins which are unseen during training in different splits of the 5-fold cross-validation set-up. On 3,162 of these proteins, AI-Bind's VecNet makes at least one false prediction, meaning that our model incorrectly labels at least one ligand as a binder which is not (false positive), or labels a ligand as non-binder which does, in fact, bind (false negative). Among these targets, we find that only 228 (5% of all the proteins) are indeed over-represented (proportions test [27]; p_{BH-fdr}[2]-value ≤ 0.05), meaning that over half the predictions involving these proteins are false. To assess the nature of these false predictions, we test their bias for false positives or false negatives. We find that 168 proteins are biased towards false positive predictions, whereas 68 are biased towards false negatives (proportions test; p_{BH-fdr}-value ≤ 0.05).

To understand whether these biases are intrinsic to the evolutionary origins of certain proteins and if AI-Bind's biases are associated with certain protein domains, we perform a phylogenetic analysis. We use MUSCLE [28], a tool for multiple protein sequence alignment, to understand the similarity between these 228 over-represented protein sequences. We observe only weak similarities between these over-represented proteins. We also reconstruct their phylogenetic tree using the neighbor-joining tree method [29] on their amino acid sequences and visualize the results using `treeio` and `ggtree` R packages [30, 31]. The results suggest that the false predictions for AI-Bind's VecNet have no bias towards any particular protein structure (Supplementary Figure 15).

Supplementary Note 11: Optimal representation of protein and ligand molecules

AI-Bind's VecNet achieves the highest inductive performance, i.e., the performance on never-before-seen proteins and ligands. VecNet uses pre-trained Mol2vec (300-dimensional) and ProtVec (100-dimensional) embeddings. These embeddings encode the structural information from the whole protein and ligand molecules [13, 14]. However, protein-ligand binding is influenced by specific molecular properties, hence we focused on the structural features that are believed to be important to binding in the literature, the so-called *engineered features* [32]. For ligands, we construct the features using the counts of different atoms in the molecule (B, Br, C, Cl, F, I, P, N, O, S), total count of atoms, count of heavy atoms, rings, hydrogen donors,

[2] p-value (Benjamini Hochberg - False Discovery Rate corrected)

hydrogen acceptors, chiral centers, molecular weight, and solubility. For proteins, we use the count of each amino acid, total number of amino acids, and sum of their overall molecular weight. In this set-up, ligands and proteins are represented using 18- and 22-dimensional features, respectively, instead of the original 300 dimensions for Mol2vec and 100 dimensions in ProtVec. Leveraging these engineered features, we observe inductive performance proximal to VecNet (Supplementary Table 5).

We further explore which dimensions of Mol2vec and ProtVec are the best in explaining the engineered features. We do so by learning matrix E through algebraic decomposition, with $VE = F$, $V \in \mathbb{R}^{N_{ligands},300}$ for ligands, and $V \in \mathbb{R}^{N_{proteins},100}$ for proteins. Matrix F encodes the engineered features: for ligands we have $F \in \mathbb{R}^{N_{ligands},18}$, while for proteins $F \in \mathbb{R}^{N_{proteins},22}$ [33]. We re-scale Mol2vec and ProtVec embeddings, as well as the engineered features, between $[0, 1]$ and perform non-negative matrix factorization to obtain E. The rows of E explain the relevance of each Mol2vec or ProtVec dimension to the engineered features. While investigating the relation between engineered features and embeddings, we observed that 15 dimensions of the 300 for Mol2Vec showed the highest variance, suggesting that relevant information is embedded in a smaller dimensional space compared to the standard dimension used in the literature. Similarly, for ProtVec we found a subset of 16 dimensions (Supplementary Figure 16). On the same note, concatenating the engineered features with Mol2vec and ProtVec does not change the inductive performance of VecNet (Supplementary Table 5). This experiment suggests that the engineered features do not add any extra information to the binding prediction task, i.e the two representations are highly correlated.

We further investigated the engineered features to understand which of them contribute most to protein-ligand binding as they have an intuitive and straightforward interpretation. SHAP [34] values show that count of carbon atoms, hydrogen acceptor count, number of chirals, count of fluorine atoms and count of oxygen atoms are the top 5 properties of a ligand that determine its binding to a protein. Presence of amino acids like Glutamic acid, Tryptophan, Asparagine, Methionine, and Threonine in a protein target, presence of aromatic rings (helps in π-stacking), presence of R groups, and N or C terminus of the protein molecules, drive protein-ligand binding (see Supplementary Table 6 and Supplementary Table 7).

VecNet with engineered features achieves a similar inductive test score as the original version. Yet, the predictions from VecNet using engineered features $\{p_{ij}^{VecNet-Engineered}\}$ show poor negative correlation with ΔG binding affinities obtained from docking simulations in the Results Section ($r_{Spearman} = -0.10$) when obtain the binary labels by thresholding using the median predicted probabilities, compared to the original VecNet with Mol2vec and ProtVec embeddings

($r_{Spearman} = -0.51$). We also observe a significant reduction in F1-score, from 0.82 to 0.64 (see Results).

Overall, when representing protein and ligand molecules in 2D, we find that only a small subset of the features drive protein-ligand binding and are able to explain the intuitive properties of the molecules. Simple molecular descriptors like the presence of R groups in the amino acids, different atom counts, charge distribution in the ligand molecule represented by hydrogen acceptor, and donor counts have significant predictive power for protein-ligand binding. However, these features do not provide insight into the surface structure of the molecules or their rigidity. Indeed, presence of binding pockets on proteins and rotatability of bonds in ligand molecules significantly influence protein-ligand binding. Including these relevant aspects in the prediction task would reduce the number of false positives, often determined by the lack of 3D structures in the model. Adding 3D features of ligands and proteins (e.g., shape of the molecules, rotation of bonds in ligand, location of binding pockets etc.) will help AI-Bind to learn the detailed mechanism behind protein-ligand binding and make more accurate predictions.

Supplementary Note 12: Random Negative Sampling

Existing binding prediction models do not consider any balancing between the binding and the non-binding pairs. In DeepPurpose, the non-binding pair generation is done by selecting random pairs of proteins and ligands which do not appear as binding pairs in the training data. As a result, an imbalance is created between the positive and negative samples for certain nodes based on their degrees in the network, and the deep learning models learn from the network topology of the protein-ligand network instead of learning the binding patterns from the molecular structures. Researchers are aware of this imbalanced training caused by binding data-sets like Tox21 and have proposed an oversampling-based approach to resolve the issue [35]. This method, however, did not improve prediction accuracy and generalizability since the root cause of degree bias is not resolved via oversampling.

In this section, we propose different methods for generating the negative pairs in a balanced fashion from the protein-ligand bipartite network. As we use a batch size of 16 in AI-Bind training [36], our ML models observe 16 data instances corresponding to a protein-ligand pair, 15 of which are negative samples, and the remaining being the positive edge.

We generate random negatives for each positive edge (t, d) representing the binding pair of target (t) and drug (d). This is achieved by randomly selecting drugs with no known binding information to t and randomly selecting proteins with no known binding information to d. A list of 15 random negative edges is generated where 7(8) random negatives relate to the target

of the positive pair and 8(7) random negatives relate to the drug of the positive pair. Since this method produces negative samples surmounting the number of positives, we use a smaller class weight for the negative samples during training.

In Supplementary Figure 17, we explore the plausibility of using the network-derived negatives for training ML models instead of the random negative samples. We show that the non-binding (or negative) degrees of the nodes in random negative sampling are correlated with the binding (or positive) degrees. Thus, the random negative samples accommodate the same topological information on the protein-ligand network as the positives, providing no additional information on the negative annotations to training. This issue is resolved by creating the network-derived negative samples, which are based on the shortest path distances in the protein-ligand bipartite network.

Finally, we studied the inductive performance of VecNet on both random negatives and network-derived negatives in a 5-fold cross-validation set-up. We observe lower inductive test performance on the random negatives (AUROC of 0.709 ± 0.011 and AUPRC of 0.566 ± 0.013) compared to the network-derived negatives (AUROC of 0.745 ± 0.032 and AUPRC of 0.729 ± 0.038).

Supplementary Note 13: Gold standard validation of binding probability profile

In this section we use gold standard protein-ligand binding data to validate the binding probability profiles predicted by AI-Bind. In other words, we validate our hypothesis that ligands bind to proteins at the valleys on the binding probability profile with high confidence gold standard experimental protein-ligand binding data [37]. This validation also shows higher propensity of the β-sheets and the coils regions to bind with the ligands.
First, we obtain the binding probability profiles generated by AI-Bind for two different ligand-protein pairs. We chose *E. Coli* protein Thymidylate Synthase. The ligands are SP-722 and SP-876. We obtain the experimentally validated secondary structure from the RCSB website, and overlay it over the binding probability profile. We then extract from the PDB file the primary binding sites of the ligand molecules. These binding locations (amino acid residues) are represented by the AC1 keyword in the PDB file. The site lists the amino acids that the ligands bind to, which are represented by red dots on the binding probability profiles (see Supplementary Figure 18). In both cases, the binding sites lie in the valleys of the probability profile, and overlay on the β-sheets and the coils regions. Supplementary Figure 19 depicts similar observations on human proteins. We have also compared the binding sites predicted by

AI-Bind with P2Rank, another state-of-the-art site detection method [38].

We compare the binding sites predicted by both AI-Bind and P2Rank to the gold-standard experimental data. For determining the binding sites from the valleys of AI-Bind's binding probability profile, we fit a sine curve to the valleys and consider the region between the points of inflection of the sine curve as the AI-Bind predicted binding site. On the other hand, P2Rank predicts the amino acid residues and the associated pockets as the binding locations. Thereafter, we compare the binding pockets predicted by AI-Bind and P2Rank to the gold standard experimental data. We observe that the AI-Bind predicted binding sites cover 64.05% of all pockets on the 195 different proteins mentioned in the gold standard dataset, whereas P2Rank is able to identify 53.57% of all of these pockets.

Supplementary References

[1] Geirhos, R. *et al.* Shortcut learning in deep neural networks. *Nat. Mach. Intell.* **2**, 665–673 (2020).

[2] Huang, K. *et al.* DeepPurpose: a deep learning library for drug–target interaction prediction. *Bioinformatics* **36**, 5545–5547 (2020).

[3] Gilson, M. K. *et al.* BindingDB in 2015: A public database for medicinal chemistry, computational chemistry and systems pharmacology. *Nucleic Acids Res.* **44**, D1045–D1053 (2015).

[4] Alstott, J., Bullmore, E. & Plenz, D. powerlaw: A python package for analysis of heavy-tailed distributions. *PLoS ONE* **9**, e85777 (2014).

[5] Barabási, A.-L. *Network Science* (Cambridge University Press, 2016).

[6] Wishart, D. S. *et al.* DrugBank: a knowledgebase for drugs, drug actions and drug targets. *Nucleic Acids Res.* **36**, D901–D906 (2007).

[7] Tang, J. *et al.* Drug target commons: A community effort to build a consensus knowledge base for drug-target interactions. *Cell Chem. Biol.* **25**, 224–229.e2 (2018).

[8] The Metabolomics Innovation Centre. FooDB. (2020) URL `http://foodb.ca`.

[9] Yannai, Shmuel. *Dictionary of Food Compounds 2nd Edition.* (CRC Press, 2012).

[10] Afendi, F. M. *et al.* KNApSAcK family databases: integrated metabolite–plant species databases for multifaceted plant research. *Plant Cell Physiol.* **53**, e1 (2012).

[11] Weininger, D. SMILES, a chemical language and information system. 1. introduction to methodology and encoding rules. *J. Chem. Inf. Comput. Sci.* **28**, 31–36 (1988).

[12] Prakash, A. B. *et al.* EigenSpokes: Surprising Patterns and Scalable Community Chipping in Large Graphs. *ICDM Workshops* p290-295 (2009).

[13] Jaeger, S., Fulle, S. & Turk, S. Mol2vec: Unsupervised machine learning approach with chemical intuition. *J. Chem. Inf. Model* **58**, 27–35 (2018).

[14] Asgari, E. & Mofrad, M. R. K. Continuous distributed representation of biological sequences for deep proteomics and genomics. *PLoS ONE* **10**, e0141287 (2015).

[15] Mikolov, T., Sutskever, I., Chen, K., Corrado, G. S. & Dean, J. Distributed representations of words and phrases and their compositionality. *Advances in neural information processing systems* **26**, 3111–3119 (2013).

[16] Rogers, D. & Hahn, M. Extended-connectivity fingerprints. *J. Chem. Inf. Model* **50**, 742–754 (2010).

[17] Doersch, C. Tutorial on variational autoencoders. Preprint at `https://arxiv.org/abs/1606.05908` (2016).

[18] Koch, G., Zemel, R. & Salakhutdinov, R. Siamese neural networks for one-shot image recognition. *In Proceedings of the 32nd International Conference on Machine Learning* **37** (Lille, France, 2015).

[19] Kingma, D. P., Salimans, T. & Welling, M. Variational dropout and the local reparameterization trick. *In Proceedings of the 28th International Conference on Neural Information Processing Systems* **2**, 2575–2583 (2015).

[20] Huang, K., Xiao, C., Glass, L. M. & Sun, J. MolTrans: Molecular interaction transformer for drug–target interaction prediction. *Bioinformatics* **37**, 830–836 (2020).

[21] Meyes, R., Lu, M., de Puiseau, C. W., & Meisen, T. Ablation studies in artificial neural networks. Preprint at `https://arxiv.org/abs/1901.08644`.

[22] Schrödinger, LLC. PyMOL molecular graphics system, Version1.8. (2015). URL `http://www.pymol.org/pymol`.

[23] Harris, T. K. & Mildvan, A. S. High-precision measurement of hydrogen bond lengths in proteins by nuclear magnetic resonance methods. *Proteins* **35**, 275–282 (1999).

[24] Laskowski, R. A., Moss, D. S & Thornton, J. M. Main-chain bond lengths and bond angles in protein structures. *J. Mol. Biol.* **231**, 1049–1067 (1993).

[25] Roth, C., Neal, B. & Lenhoff, A. Van der waals interactions involving proteins. *Biophys. J.* **70**, 977–987 (1996).

[26] Bajusz, D., Rácz, A. & Héberger, K. Why is tanimoto index an appropriate choice for fingerprint-based similarity calculations?. *J. Cheminform.* **7**, 20 (2015).

[27] Newcombe, R. G. Two-sided confidence intervals for the single proportion: comparison of seven methods. *Stat. Med.* **17**, 857–572 (1998).

[28] Papadopoulos, J. S. & Agarwala, R. COBALT: constraint-based alignment tool for multiple protein sequences. *Bioinformatics* **23**, 1073–1079 (2007).

[29] Edgar, R. C. MUSCLE: multiple sequence alignment with high accuracy and high throughput. *Nucleic Acids Res.* **32**, 1792–1797 (2004).

[30] Yu, G. Using ggtree to visualize data on tree-like structures. *Curr. Protoc. Bioinformatics* **69**, e96 (2020).

[31] Wang, L.-G. *et al.* Treeio: An r package for phylogenetic tree input and output with richly annotated and associated data. *Mol. Biol. Evol.* **37**, 599–603 (2019).

[32] Rohrer, S. G. & Baumann, K. Maximum unbiased validation (MUV) data sets for virtual screening based on PubChem bioactivity data. *J. Chem. Inf. Model* **49**, 169–184 (2009).

[33] Henderson, K. *et al.* Rolx: structural role extraction & mining in large graphs.. *In Proceedings of the 18th ACM SIGKDD international conference on Knowledge discovery and data mining*, p1231–1239 (2012).

[34] Lundberg, S. M. & Lee, S.-I. A unified approach to interpreting model predictions. *Advances in Neural Information Processing Systems* **30** (Curran Associates, Inc., 2017).

[35] Idakwo, G. *et al.* Structure–activity relationship-based chemical classification of highly imbalanced Tox21 datasets. *J. Cheminform.*, **12**, 66 (2020).

[36] Bengio, Y. Practical recommendations for gradient-based training of deep architectures. Preprint at `https://arxiv.org/abs/1206.5533` (2012).

[37] Cheng, T., Li, X., Li, Y., Liu, Z. & Wang, R. Comparative assessment of scoring functions on a diverse test set. *J. Chem. Inf. Model* **49**, 1079–1093 (2009).

[38] Kriválk, R. & Hoksza, D. P2Rank: machine learning based tool for rapid and accurate prediction of ligand binding sites from protein structure. *J. Cheminform.* **10**, 39 (2018).

Supplementary Table 1: **Transductive test performance of a duplex configuration model.** The model has unipartite layers with varying annotation distribution $P(k)$ and correlation $r_{Spearman}(k, K_\mathrm{d})$. The network has $N = 1{,}507$ nodes, the same as the number of unique proteins in the BindingDB training data. The constrained features are consistent with the protein sample in BindingDB, e.g., for the power law network we use the degree sequence derived from the protein network in BindingDB data, while the Poisson network has the same average degree $\langle k \rangle = 47$ of the power law network. To achieve $r_{Spearman}(k, K_\mathrm{d}) \approx 0$, we shuffle the edges of the original network, which removes the anti-correlation between k and $\mathrm{K_d}$.

Fluctuations in K_d	Annotation distribution	$r_{Spearman}(k, K_\mathrm{d})$	p_{bind}	AUROC	AUPRC
Strictly no fluctuation	Power law	≈ -1.0	0.16	0.94	0.89
No fluctuation	Power law	≈ -0.47	0.16	0.95	0.89
Constant fluctuations	Power law	≈ -0.47	0.16	0.64	0.26
Varying fluctuations	Power law	≈ -0.47	0.16	0.86	0.59
Strictly no fluctuation	Poisson	≈ -1.0	0.16	0.92	0.86
No fluctuation	Poisson	≈ -0.47	0.16	0.95	0.88
Constant fluctuations	Poisson	≈ -0.47	0.16	0.69	0.30
Varying fluctuations	Poisson	≈ -0.47	0.16	0.72	0.33
Strictly no fluctuation	Power law	≈ 0	0.16	0.49	0.15
No fluctuation	Power law	≈ 0	0.16	0.51	0.16
Constant fluctuations	Power law	≈ 0	0.16	0.50	0.16
Varying fluctuations	Power law	≈ 0	0.16	0.49	0.14
Strictly no fluctuation	Poisson	≈ 0	0.16	0.51	0.16
No fluctuation	Poisson	≈ 0	0.16	0.50	0.15
Constant fluctuations	Poisson	≈ 0	0.16	0.51	0.17
Varying fluctuations	Poisson	≈ 0	0.16	0.51	0.17

Supplementary Table 2: **Effects of path length.** As the path length to a fixed protein i increases, the mean and variance of the length of the low-dimensional embedding of the ligand $\|\bar{u}_j\|$ decrease.

Path Length i to j	1	3	5	7	9	11	13
Mean $\|\bar{u}_j\|$	0.045	0.035	0.014	0.004	0.001	0.0001	$5 \cdot 10^{-7}$
Std. dev. $\|\bar{u}_j\|$	0.075	0.035	0.025	0.014	0.008	0.001	$4 \cdot 10^{-6}$

Supplementary Table 3: **Results across different models.** We summarize all performances on the network-derived negative samples. We perform 5-fold cross-validation, reporting AUROC and AUPRC averaged over the 5 runs with random initialization and data split. Results are reported separately on 3 different train-validation-test splits with different data held out in the validation and testing sets: (1) **Unseen edges (Transductive test)** - test sets contain unseen edges in the train network, (2) **Unseen targets (Semi-inductive test)** - test sets contains pairs with proteins that do not appear in train or validation set, (3) **Unseen nodes (Inductive test)** - nodes in test set pairs are completely disjoint from the training set. *Random Input Tests:* We train and test AI-Bind's VecNet replacing node features with random entries drawn from a uniform distribution in the range $U([-1, 1]^d)$. We run two tests (1) Unif. - All node features are replaced by vectors drawn from a uniform distribution $U([-1, 1]^d)$, (2) Unif.Targ. - Only the target node features are replaced by vectors from $U([-1, 1]^d)$; drug features remain the same. Note that the transductive (unseen edges) performance is reported based on the models optimized for unseen nodes, except for the case of the Random Inputs, where we report performance based on models optimized for unseen targets.

Model	Test Data Division					
	Transd.		Semi-induc.		Induc.	
	AUROC	AUPRC	AUROC	AUPRC	AUROC	AUPRC
Configuration	$.738 \pm .015$	$.734 \pm .027$	$.748 \pm .022$	$.688 \pm .036$	$.500 \pm .000$	$.464 \pm .017$
VecNet	$.796 \pm .011$	$.814 \pm .021$	$.776 \pm .025$	$.760 \pm .039$	$.750 \pm .032$	$.718 \pm .029$
Siamese	$.656 \pm .033$	$.638 \pm .004$	$.664 \pm .018$	$.620 \pm .045$	$.642 \pm .029$	$.582 \pm .036$
VAENet	$.774 \pm .015$	$.714 \pm .050$	$.758 \pm .018$	$.714 \pm .034$	$.740 \pm .014$	$.702 \pm .033$
DeepPurpose	$.772 \pm .019$	$.800 \pm .019$	$.644 \pm .021$	$.590 \pm .012$	$.646 \pm .023$	$.576 \pm .009$
Random Inputs						
VecNet Unif.	$.668 \pm .013$	$.708 \pm .008$	$.534 \pm .021$	$.548 \pm .013$	$.456 \pm .048$	$.466 \pm .038$
VecNet - Targ.	$.706 \pm .011$	$.718 \pm .011$	$.566 \pm .038$	$.562 \pm .026$	$.556 \pm .011$	$.501 \pm .029$

Supplementary Table 4: **Comparing AI-Bind with MolTrans.** We compare transductive, semi-inductive, and inductive performances of MolTrans with AI-Bind's VecNet. MolTrans uses a combination of a sub-structural pattern mining algorithm, an interaction modeling module, and an augmented transformer encoder to better learn the molecular structures. VecNet performs better compared to MolTrans in semi-inductive and inductive tests. This analysis validates that unsupervised pre-training improves the generalizability of the protein-ligand binding models. We have trained and tested MolTrans on both BindingDB (used in the original paper) and network-derived negatives (AI-Bind data).

Model	Test Data Division					
	Transd.		Semi-induc.		Induc.	
	AUROC	AUPRC	AUROC	AUPRC	AUROC	AUPRC
MolTrans[a]	$.950 \pm .041$	$.888 \pm .087$	$.652 \pm .050$	$.404 \pm .059$	$.572 \pm .104$	$.432 \pm .105$
MolTrans[b]	$.878 \pm .082$	$.804 \pm .046$	$.646 \pm .018$	$.498 \pm .054$	$.612 \pm .028$	$.478 \pm .034$
DeepPurpose	$.772 \pm .019$	$.800 \pm .019$	$.642 \pm .020$	$.592 \pm .029$	$.646 \pm .022$	$.582 \pm .027$
VecNet	$.796 \pm .011$	$.814 \pm .021$	$.800 \pm .021$	$.754 \pm .042$	$.748 \pm .033$	$.728 \pm .028$

[a]BindingDB data
[b]Network-derived Negatives

Supplementary Table 5: **Optimal feature selection.** We observe that AI-Bind's VecNet shows similar performances in inductive tests when Mol2vec and ProtVec are replaced by simple engineered features encoding certain properties of protein and ligand molecules. Furthermore, we observe that only 15 dimensions of Mol2vec and 16 dimensions of ProtVec embeddings encode these molecular properties driving the binding task. Using these feature subsets of Mol2vec and ProtVec helps VecNet achieve similar inductive performance. Concatenating the engineered features with Mol2vec and ProtVec does not improve inductive performance. This suggests that the information encoded in the engineered features strongly correlates with Mol2vec and ProtVec embeddings.

Performance	Original	Engineered features	Mol2vec and ProtVec dimensions explaining Engineered Features	Concatenated: Mol2vec/ProtVec + Engineered features
AUROC	0.742 ± 0.031	0.740 ± 0.028	0.726 ± 0.092	0.730 ± 0.040
AUPRC	0.742 ± 0.034	0.740 ± 0.041	0.726 ± 0.040	0.730 ± 0.045

Supplementary Table 6: **Engineered feature importance for ligands.** We tabulate the engineered features in descending order of average absolute SHAP importance over AI-Bind data. A higher SHAP value represents more relevance of the molecular property in predicting protein-ligand binding.

Feature	Average SHAP Importance
Count of Carbon Atom	0.012546
Hydrogen Acceptor Count	0.012362
Number of Chirals	0.008750
Count of Flourine Atoms	0.006527
Count of Oxygen Atoms	0.006184
Hydrogen Donor Count	0.005647
Number of Atoms	0.004165
Count of Heavy Atoms	0.003468
Solubility in Water ($\log p$)	0.003202
Molecular Weight	0.003161
Count of Nitrogen Atoms	0.002007
Count of Chlorine Atoms	0.001720
Count of Sulphur Atoms	0.001483
Number of Rings	0.000525
Count of Phosphorus Atoms	0.000191
Count of Iodine Atoms	0.000130
Count of Bromine Atoms	0.000083
Count of Boron Atoms	0.000070

Supplementary Table 7: **Engineered feature importance for proteins.** We tabulate the engineered features in descending order of average absolute SHAP importance over AI-Bind data. A higher SHAP value represents more relevance of the molecular property in predicting protein-ligand binding.

Feature	Average SHAP Importance
Count of Glutamic acid (E)	0.036747
Count of Tryptophan (W)	0.033210
Count of Asparagine (N)	0.024770
Count of Methionine (M)	0.022734
Count of Threonine (T)	0.021194
Count of Glycine (G)	0.020832
Count of Arginine (R)	0.019599
Count of Phenylalanine (F)	0.017040
Count of Cysteine (C)	0.016428
Count of Isoleucine (I)	0.016215
Count of Alanine (A)	0.015732
Count of Histidine (H)	0.014813
Count of Leucine (L)	0.014026
Count of Tyrosine (Y)	0.013844
Count of Proline (P)	0.013014
Count of Valine (V)	0.011152
Count of Serine (S)	0.010930
Count of Lysine (K)	0.008689
Count of Aspartic acid (D)	0.008303
Total amino acid count	0.003381
Count of Glutamine (Q)	0.002957
Molecular Weight	0.002088

Supplementary Table 8: **List of Proteins Docked.** When validating AI-Bind, we performed docking simulations on 146 protein-ligand interactions within the top 100 and bottom 100 of the AI-Bind predictions for SARS-CoV-2. The 3D structures for the proteins were collected from the associated PDB codes. The number of ligands docked to each is reported. The full list of interactions used docking simulations with Ligand names can be found in our repository on Zenodo.

GENE	PDB Code	Number of Ligands Docked
ARF6	2A5F	1
ATP1B1	7D91	1
BAG5	3A8Y	1
BMPR1B	3MDY	1
CDK5RAP2	6X0V	1
CEP250	6OQA	11
CLIP4	2Z0W	1
FAM98A	AF_AFQ8NCA5F1	11
GNB1	5UZ7	1
GOLGA2	6IW8	1
GORASP1	4REY	1
GRIA2	2WJW	37
ITGB1	3G9W	4
LARP1	5V4R	1
MRPS2	6RW5	1
POR	1B1C	1
PRKACA	2GU8	1
PTPN22	2P6X	52
PVR	3UDW	1
RALA	2A9K	1
ROR2	3ZZW	1
SNIP1	5Z56	1
TEC	2LUL	7
TIMM10	2BSK	1
TIMM10B	7CGP	1
TIMM29	7CGP	2
TLE1	1GXR	1
TUBGCP3	6V6B	2

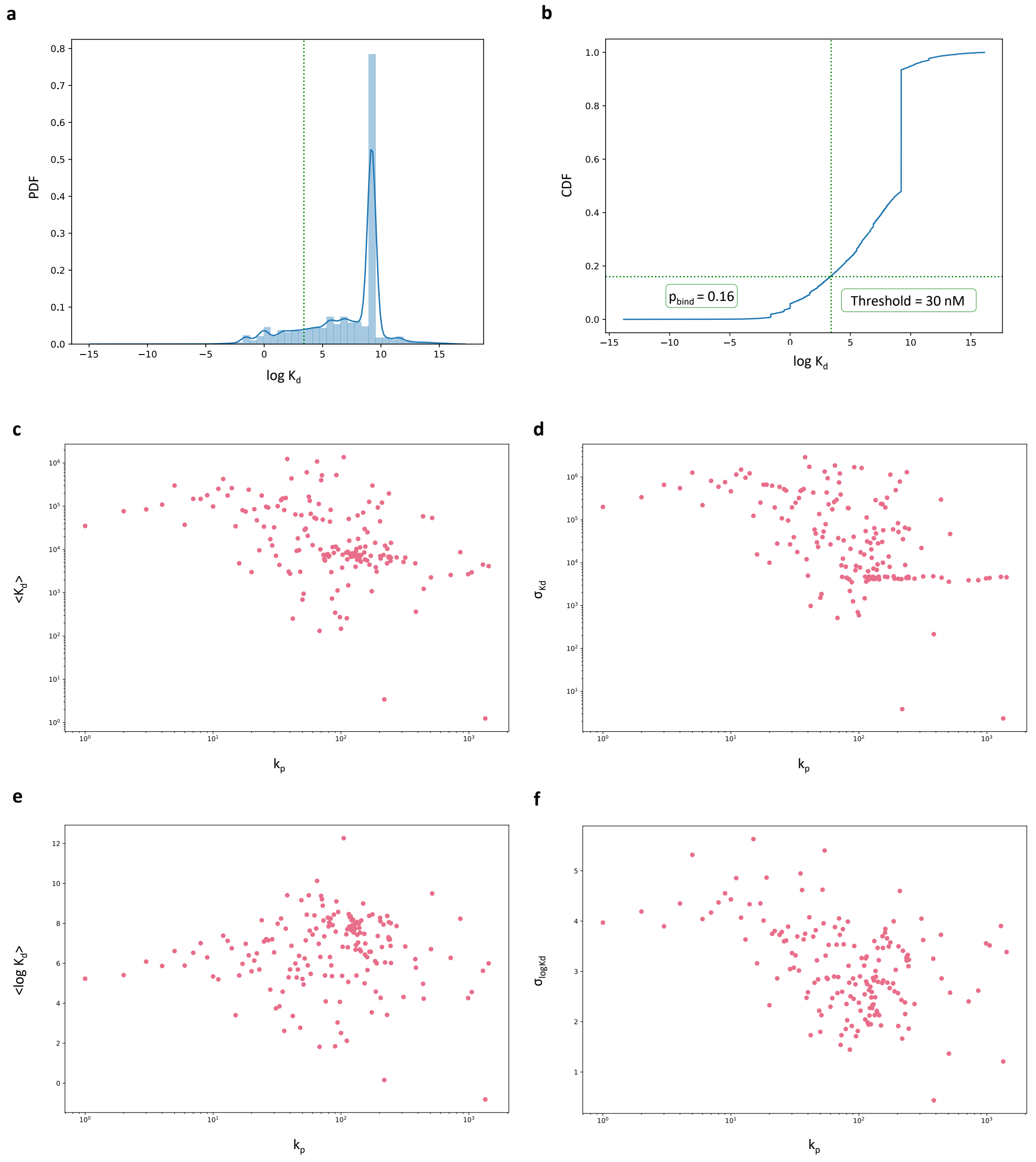

Supplementary Figure 1: **Disassociation constant $\mathrm{K_d}$ and its relation with the number of annotations/records k in BindingDB. (a)-(b)** Density distribution and cumulative distribution of log $\mathrm{K_d}$ in BindingDB training data. With threshold $30nM$, we obtain an average binding probability of $p_{bind} = 0.16$. **(c)-(f)** The averages and the standard deviations for $\mathrm{K_d}$ in both linear and log spaces are calculated over the links associated with the nodes with a given degree k. We do not observe a significant correlation between k and $\langle \log \mathrm{K_d} \rangle$, as opposed to the anti-correlation observed in the linear space, but we do find that k anti-correlates with the standard deviation of log K_d with $r_{Spearman}(k, \sigma_{\log K_d}) = -0.38$. Similarly, in the linear space we observe $r_{Spearman}(k, \sigma_{K_d}) = -0.51$. This observation implies that the lower degree nodes have higher fluctuations in the associated log $\mathrm{K_d}$ values compared to the higher degree nodes. The Source Data File provided with the manuscript contains the number of samples per data point in the plots.

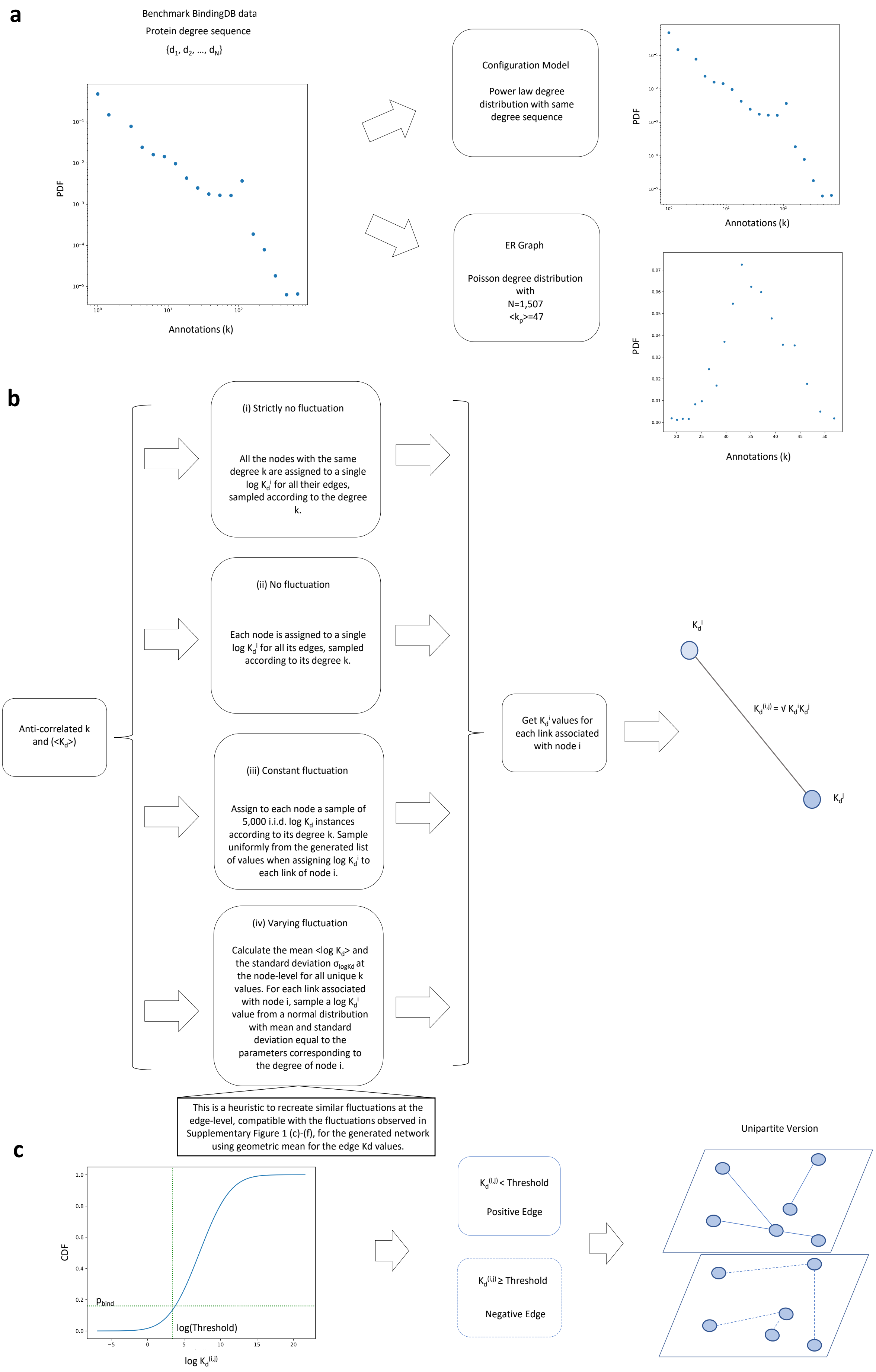
a
Benchmark BindingDB data
Protein degree sequence
{d1, d2, ..., dN}
PDF
Annotations (k)
Configuration Model
Power law degree distribution with same degree sequence
ER Graph
Poisson degree distribution with
N=1,507
<kp>=47
PDF
Annotations (k)
PDF
Annotations (k)
b
Anti-correlated k and (<Kd>)
(i) Strictly no fluctuation
All the nodes with the same degree k are assigned to a single log Kdi for all their edges, sampled according to the degree k.
(ii) No fluctuation
Each node is assigned to a single log Kdi for all its edges, sampled according to its degree k.
(iii) Constant fluctuation
Assign to each node a sample of 5,000 i.i.d. log Kd instances according to its degree k. Sample uniformly from the generated list of values when assigning log Kdi to each link of node i.
(iv) Varying fluctuation
Calculate the mean <log Kd> and the standard deviation σlogKd at the node-level for all unique k values. For each link associated with node i, sample a log Kdi value from a normal distribution with mean and standard deviation equal to the parameters corresponding to the degree of node i.
This is a heuristic to recreate similar fluctuations at the edge-level, compatible with the fluctuations observed in Supplementary Figure 1 (c)-(f), for the generated network using geometric mean for the edge Kd values.
Get Kdi values for each link associated with node i
Kdi
Kd(i,j) = √ KdiKdj
Kdj
c
CDF
pbind
log(Threshold)
log Kd(i,j)
Kd(i,j) < Threshold
Positive Edge
Kd(i,j) ≥ Threshold
Negative Edge
Unipartite Version

Supplementary Figure 2: **Experimental set-up for studying the emergence of topological shortcuts.** **(a)** We generate random unipartite networks inspired by the topological and kinetic features of the protein sample in BindingDB. In particular, we fix the size of the network to $N = 1,507$ and use the same degree sequence as in BindingDB, while for the Poisson case, the link density is constrained by the average number of annotations in the power law network. **(b)** We explore four different strategies for sampling the kinetic constants constituting different fluctuation scenarios in $\mathrm{K_d}$ values at the link level: (i) sampling without any variance in the $\log \mathrm{K_d}^i$ values contributed by node i to its links, where the sampled $\log \mathrm{K_d}^i$ values are selfsame for all nodes i with a given degree k, (ii) sampling without any variance in the $\log \mathrm{K_d}^i$ values contributed by node i to its links in presence of node-level fluctuations for the sampled $\log \mathrm{K_d}^i$, for nodes with the same degree k, (iii) sampling with variance in the $\log \mathrm{K_d}^i$ values, the variance being independent of the degree k of node i and equal to logarithmic variance of $\log \mathrm{K_d}$ in the BindingDB protein sample, and (iv) sampling with variance in the $\log \mathrm{K_d}^i$ values, the variance decreasing as a function of k, replicating the observation in BindingDB data.
According to the sampling strategy, each node contributes to all its edges with a different extent of variability. The final disassociation constant $\mathrm{K_d}^{(i,j)}$ assigned to edge (i, j) is the geometric mean of the contribution $\mathrm{K_d}^i$ from node i and the contribution $\mathrm{K_d}^j$ from node j. In scenarios (i) and (ii) each node is assigned to a single $\log \mathrm{K_d}^i$ for all its edges, sampled according to its degree k. In (iii) we follow a similar approach to (ii), but instead of sampling a single value, we assign to each node a sample of 5,000 i.i.d. $\log \mathrm{K_d}$ instances. Thereafter, when assigning $\log \mathrm{K_d}^i$ to each link associated with node i, we sample uniformly from the generated list of values. In scenario (iv), to generate a final network with edge-level $\mathrm{K_d}$ fluctuations compatible with real data observations in Supplementary Figures 1c-f, we leverage as input the observed $\mathrm{K_d}$ fluctuations at node level per degree k. Indeed, this strategy is a good heuristic to generate networks compatible with BindingDB data, given the subsequent error propagation due to the edge-level $\mathrm{K_d}$ computation using the geometric mean. Specifically, we first calculate the mean $\langle \log \mathrm{K_d} \rangle$ and the standard deviation $\sigma_{\log K_d}$ for all unique k values at the node level. Then, for each link associated with node i we sample a $\log \mathrm{K_d}^i$ value from a normal distribution with mean and standard deviation equal to the parameters corresponding to the degree of node i.
In the uncorrelated scenario, we randomly shuffle the $\mathrm{K_d}$ values associated with the links, which removes the anti-correlation between k and $\mathrm{K_d}$.
(c) We select as threshold for $\mathrm{K_d}^{(i,j)}$ the value for which a fixed percentage of the annotations become positive or binding, enforcing the constraint on the observed $p_{bind} = 0.16$. Based on this threshold, we generate the duplex layers with positive and negative edges and calculate the multilink degree sequences, input to the duplex configuration model (see Methods).

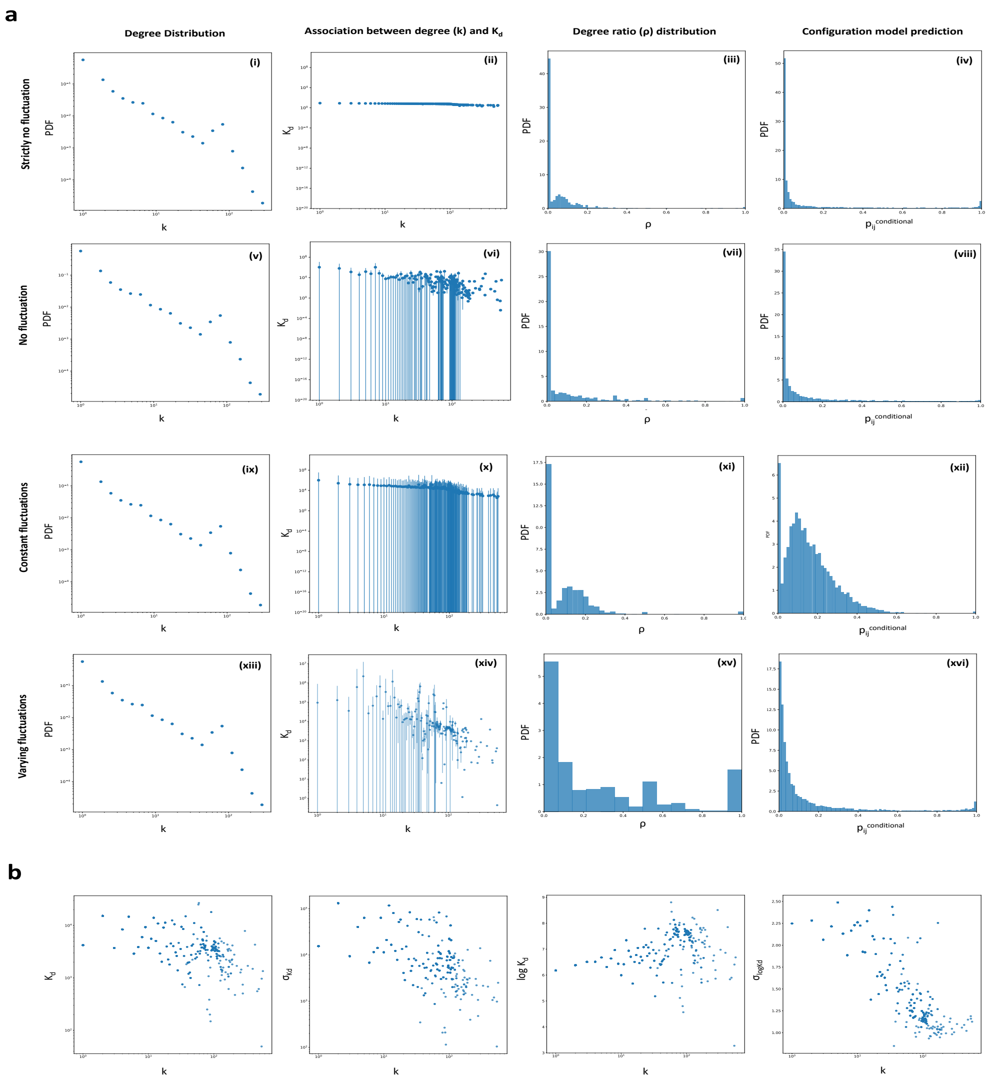

Supplementary Figure 3: **Emergence of topological shortcuts in scale-free networks.** **(a)** In the first two scenarios with "strictly no fluctuation" and "no fluctuation", where link-level fluctuations are absent in $\mathrm{K_d}$ values for a given degree k, the relation between k and $\mathrm{K_d}$ is monotonic and the configuration model is able to predict the link types using only the degree information of the nodes. In the third scenario with "constant fluctuations", link-level fluctuations are introduced in $\mathrm{K_d}$ values for a given k, and the monotonicity of the relation between k and $\mathrm{K_d}$ is disrupted. Thus, the configuration model cannot learn the link types only by using the degree information. The fourth scenario with "varying fluctuations" is compatible with the observations in BindingDB. Although k is no longer a good predictor of the link-level $\mathrm{K_d}$ values, the extent of the $\mathrm{K_d}$ fluctuations is significantly smaller for the hubs, making the link classification task easier. Since the majority of the links in the protein-ligand interaction network are associated with the hubs and are present both in train and test datasets under a random edge split, we observe the configuration model achieving excellent transductive test performance by learning only the degree sequence, and recreating the observed topological shortcuts in DeepPurpose. Note that we impose the same range for the y-axis in the k vs $\mathrm{K_d}$ plots to enhance the comparability across different scenarios. While this stylistic choice works well for panels (vi)-(x)-(xiv), for panel (ii) is misleading, as the strict anti-correlation between k and $\mathrm{K_d}$ with $r_{Spearman}(k, \langle \mathrm{K_d} \rangle) = -1$ appears as constant. **(b)** In the scenario with "varying fluctuations", generated by following the heuristic explained in Supplementary Figure 2-b, the link level fluctuations in $\mathrm{K_d}$ for a given value of k resemble the observations for BindingDB data shown in Supplementary Figures 1c-f.

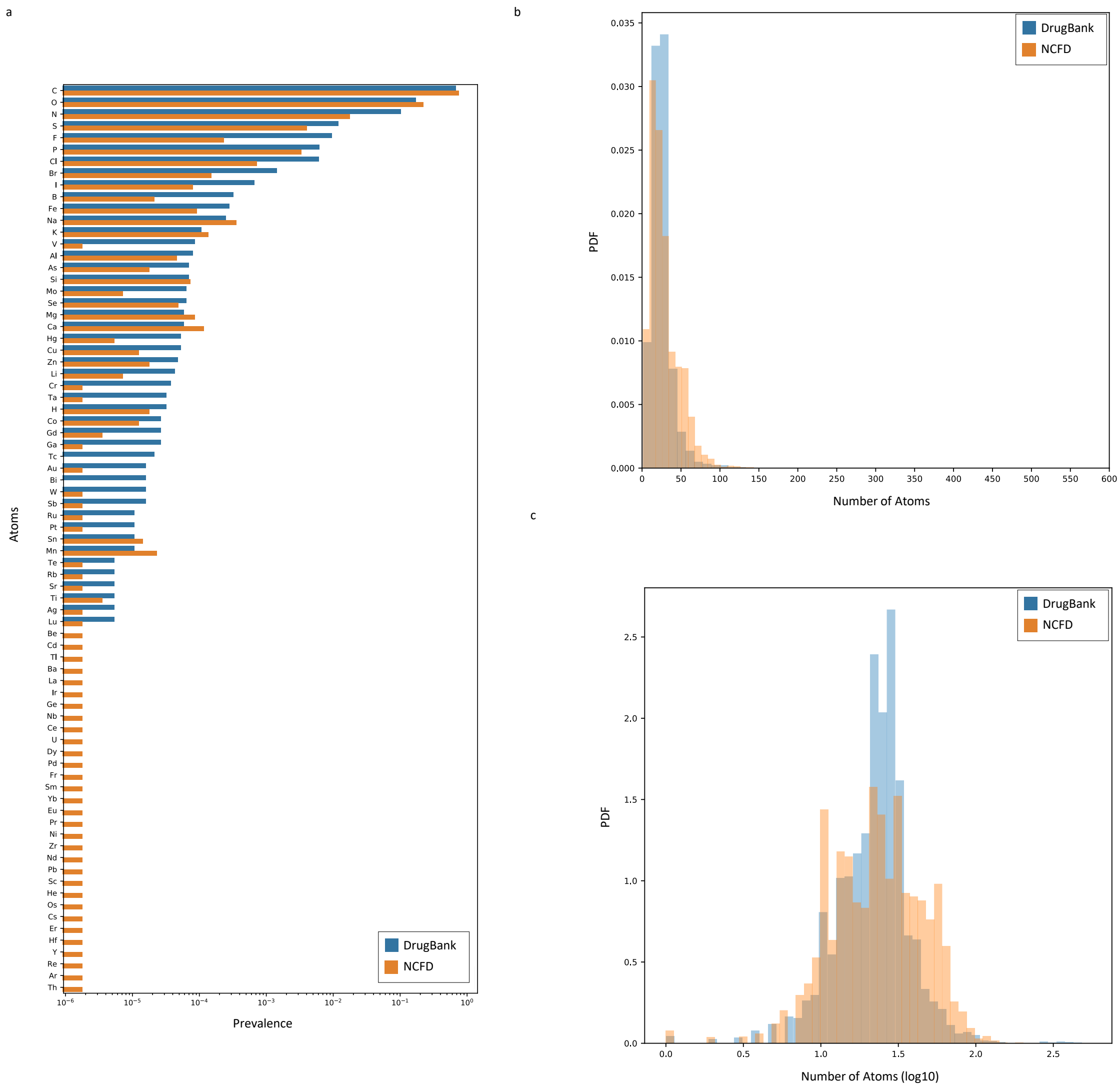

Supplementary Figure 4: **Naturally occurring compounds are structurally more complex than drugs. (a)** Prevalence of different atoms in DrugBank and natural ligands present in NCFD. Natural ligands show more diversity in terms of the constituent atoms. **(b)** The distribution of the number of atoms across the ligand molecules in DrugBank and NCFD, and **(c)** The distribution of the number of atoms in $\log_{10}$ across the ligand molecules in DrugBank and NCFD.

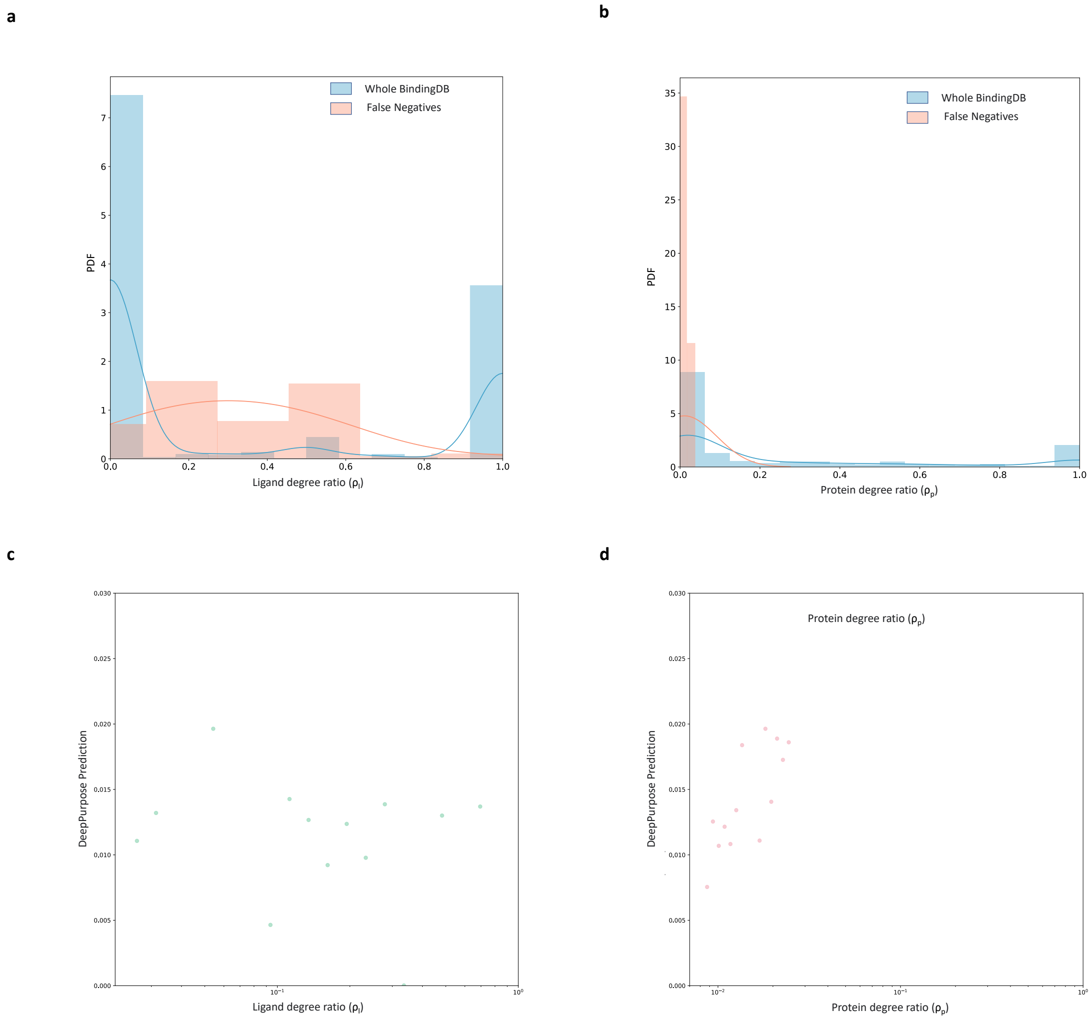

Supplementary Figure 5: **Annotation bias in top 100 false negative predictions made by DeepPurpose.** **(a)-(b)** Degree ratio distribution of the nodes involved in the false negative predictions is shown compared to all the nodes in the BindingDB data. The false negative predictions correspond to proteins and ligands with low degree ratios. **(c)-(d)** DeepPurpose predicts lower binding probabilities for the nodes with lower degree ratios.

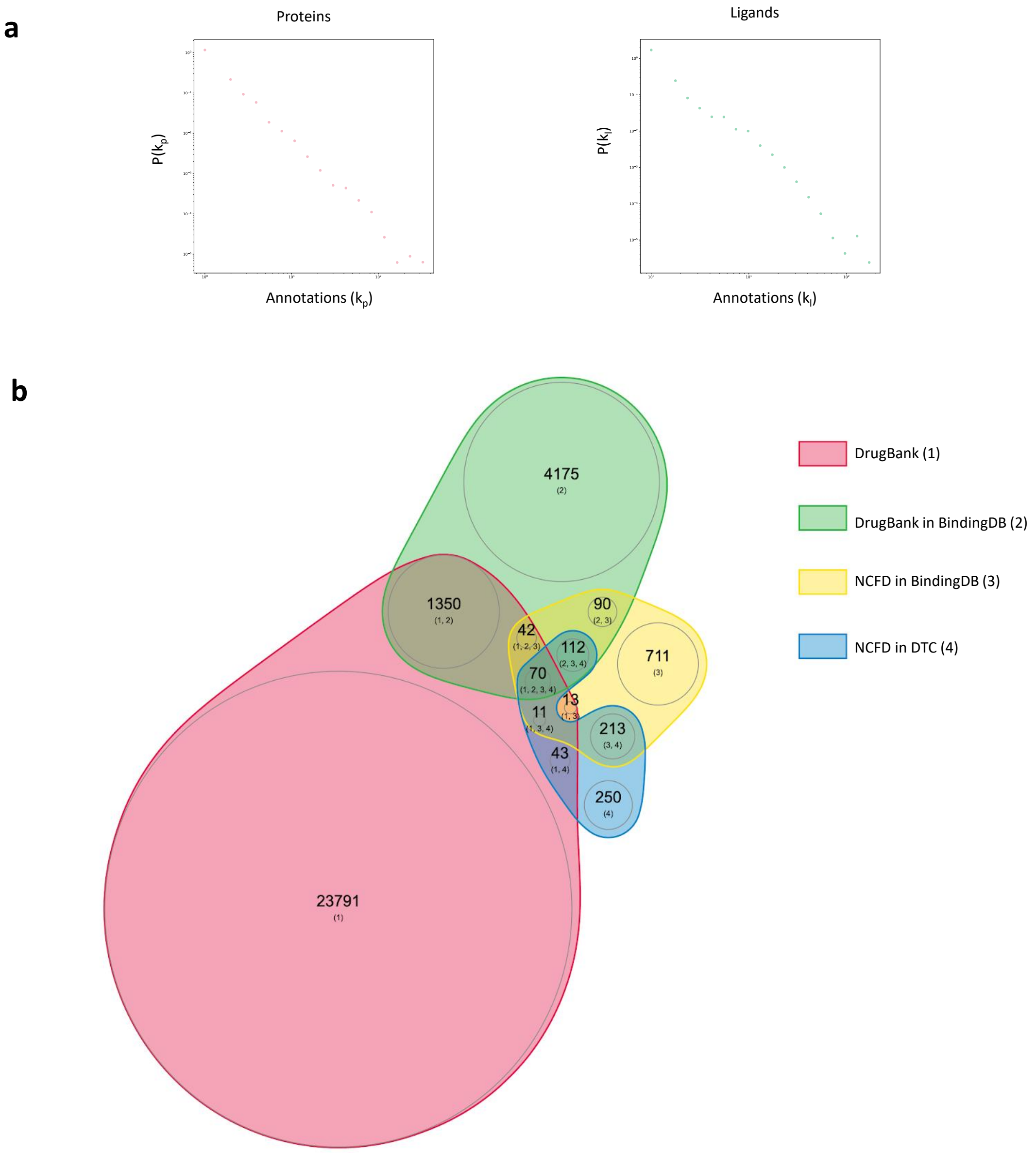

Supplementary Figure 6: **Network property of the DrugBank DTI and Venn diagram of positive binding samples across different databases. (a)** Annotation distribution of the proteins and the drugs in DrugBank are fat-tailed. The nature of the annotation distribution is similar to our observations in BindingDB. **(b)** AI-Bind training data combines protein-ligand binding data from three databases: DrugBank, BindingDB, and Drug Target Commons (DTC). The majority of the binding examples are taken from DrugBank. BindingDB and DTC are used to obtain additional protein-ligand pairs, especially to maximize the binding information involving naturally occurring ligands.

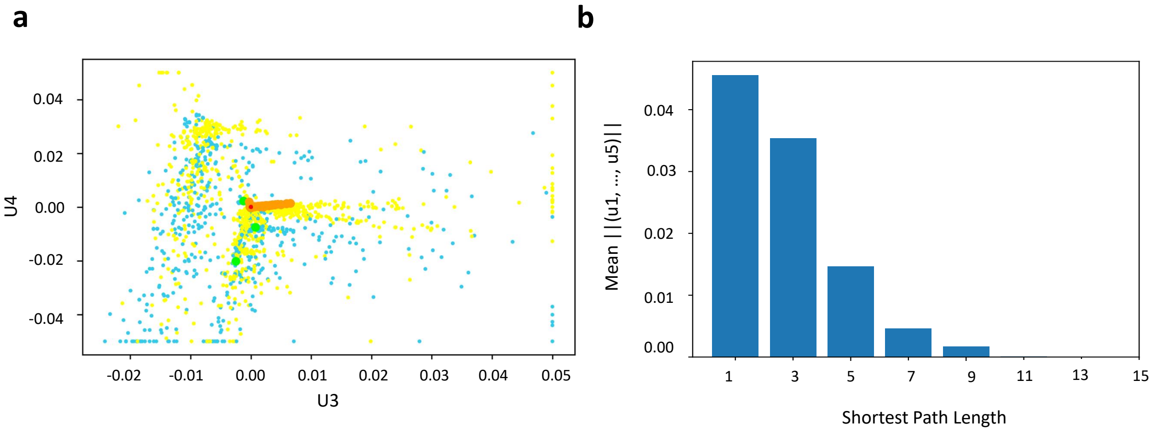

Supplementary Figure 7: **EigenSpokes Analysis.** **(a)** Network-based dimension reduction of nodes in the full protein-ligand network. Node i is represented by the vector $\bar{u}_i = (u_1, u_2, u_3, u_4, u_5) \in \mathbb{R}^5$. Here we visualize (u_3, u_4) for only the ligands. Coloring is based on the hop-distances from an example target BPT4: Green = 1 hop, Blue = 3 hops, Yellow = 5 hops, Orange = 7 hops, Red $\geq$ 9 hops. We see that at $>$ 7 hop, most nodes are very close to the origin. **(b)** Mean of all reduced vector magnitudes $\|\bar{u}_j\|$ averaged over all pairs (i, j) of a given path length. We see a significant decrease in magnitude as the shortest path length increases.

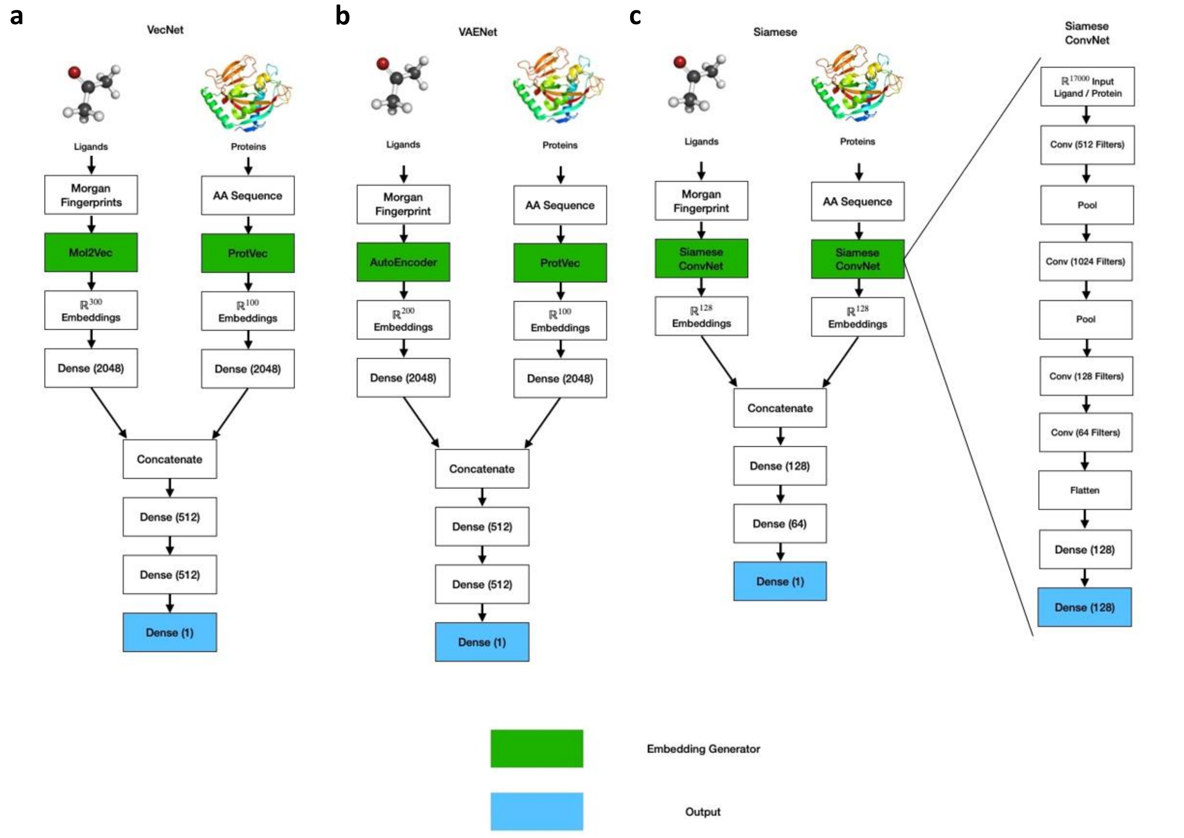

Supplementary Figure 8: **Deep architectures of VecNet, VAENet, and Siamese model.** **(a)** VecNet uses Mol2vec and ProtVec as the unsupervised pre-trained models for ligand and protein embeddings respectively. The dense layers act as decoders, and are trained using the network-derived dataset. **(b)** VAENet architecture is similar to VecNet, where Mol2vec embeddings are replaced with embeddings obtained from a variational auto-encoder. This auto-encoder is trained on ≈ 9.5 million compounds from the ZINC database. **(c)** Siamese model embeds both proteins and ligands onto the same latent space. Siamese ConvNet blocks minimize the triplet loss between the proteins binding to the same ligand. We follow a similar approach for generating the ligand embeddings.

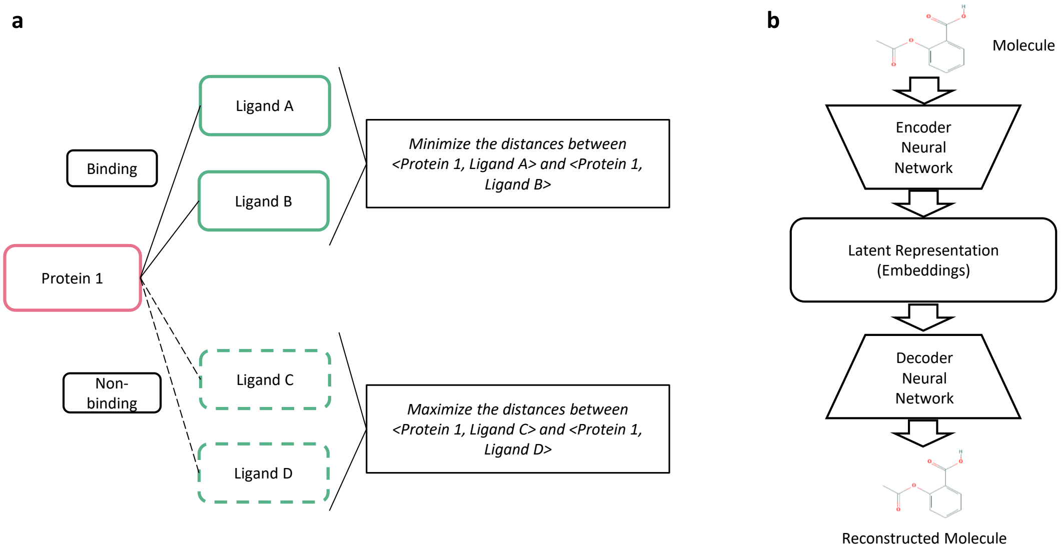

Supplementary Figure 9: **Logical flow of the Siamese Model and Variational Auto-Encoder**. **(a)** We minimize the embedded Euclidean distances between the proteins which bind to the same ligand, and maximize the distance between the non-binding ones. Similar logic is applied for creating the ligand embeddings. **(b)** Variational auto-encoder minimizes the reconstruction loss for the ligands to create a latent space embedding. We generate Morgan fingerprints from the isomeric SMILES and feed that to the auto-encoder. The auto-encoder generates latent space representations by minimizing reconstruction loss on the fingerprints.

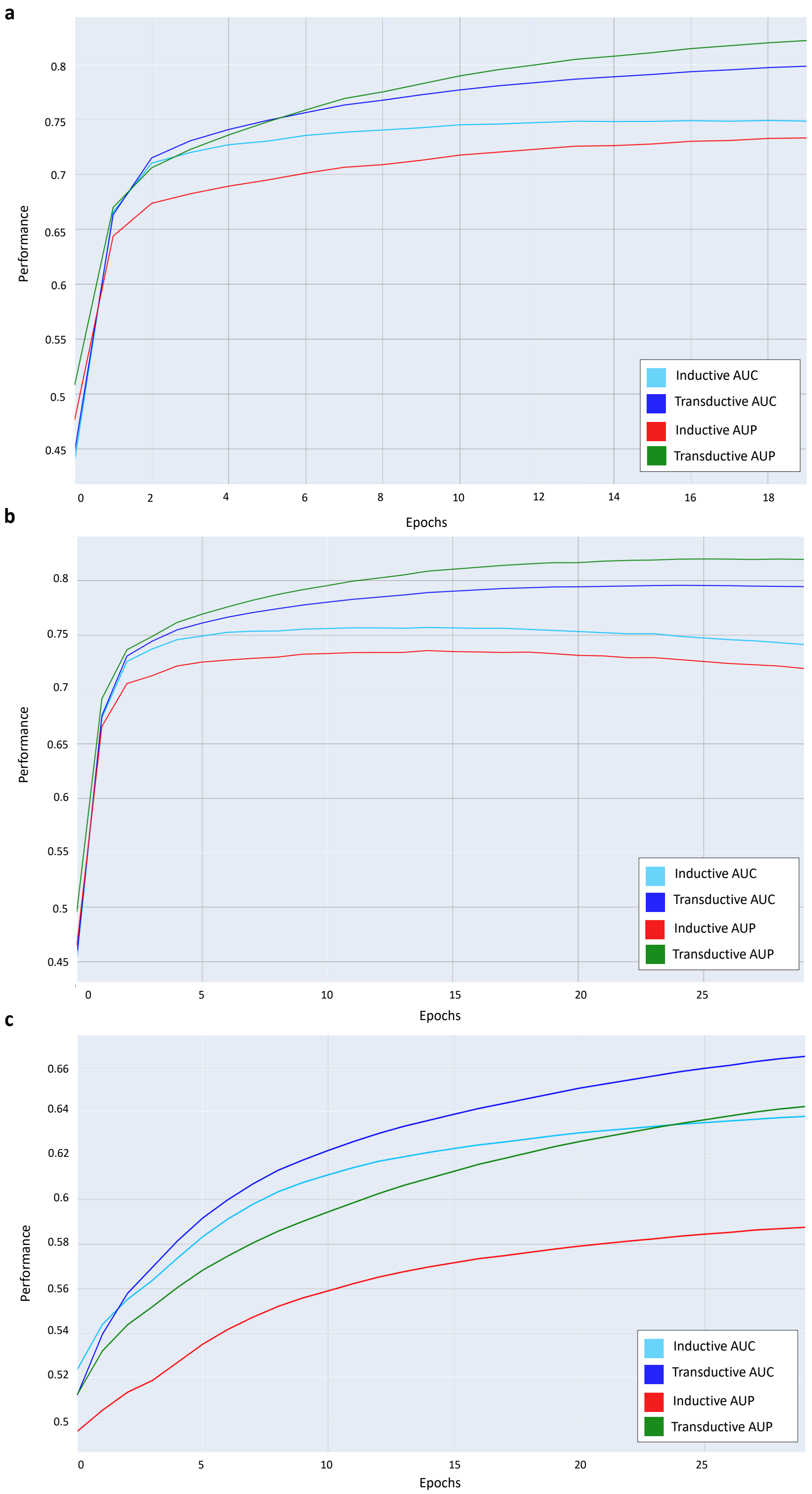

Supplementary Figure 10: **Training curves for three AI-Bind architectures.** We plot the training curves for **(a)** VecNet, **(b)** VAENet, and **(c)** Siamese model over 30 epochs. The AUROC and the AUPRC are separately shown for the transductive (unseen edges) and inductive (unseen nodes) test scenarios.

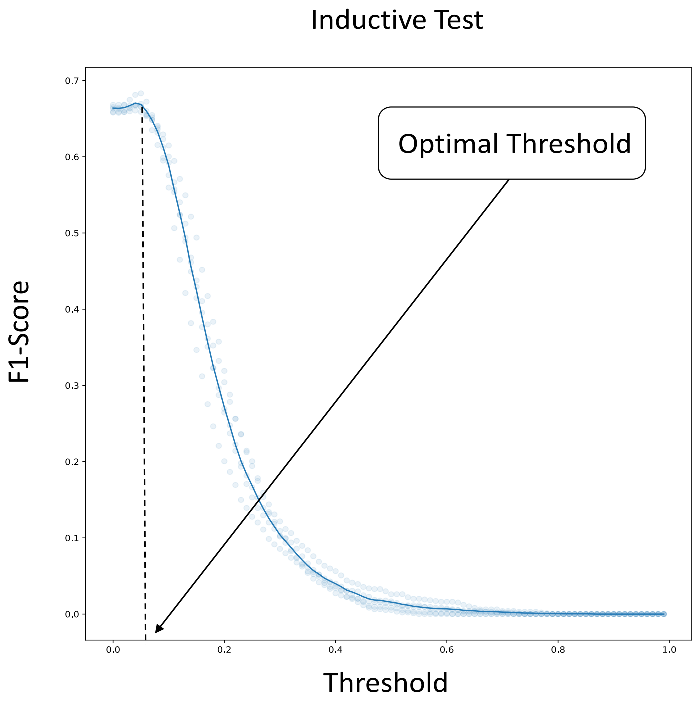

Supplementary Figure 11: **F1-Score and Optimal Threshold.** We plot the F1-scores for the trained VecNet model relative to the classification threshold in the inductive test scenario. The threshold value corresponding to the highest F1-score is considered as the optimal threshold, and is used to obtain the binary labels from the predicted binding probabilities. We obtain an optimal threshold of 0.09.

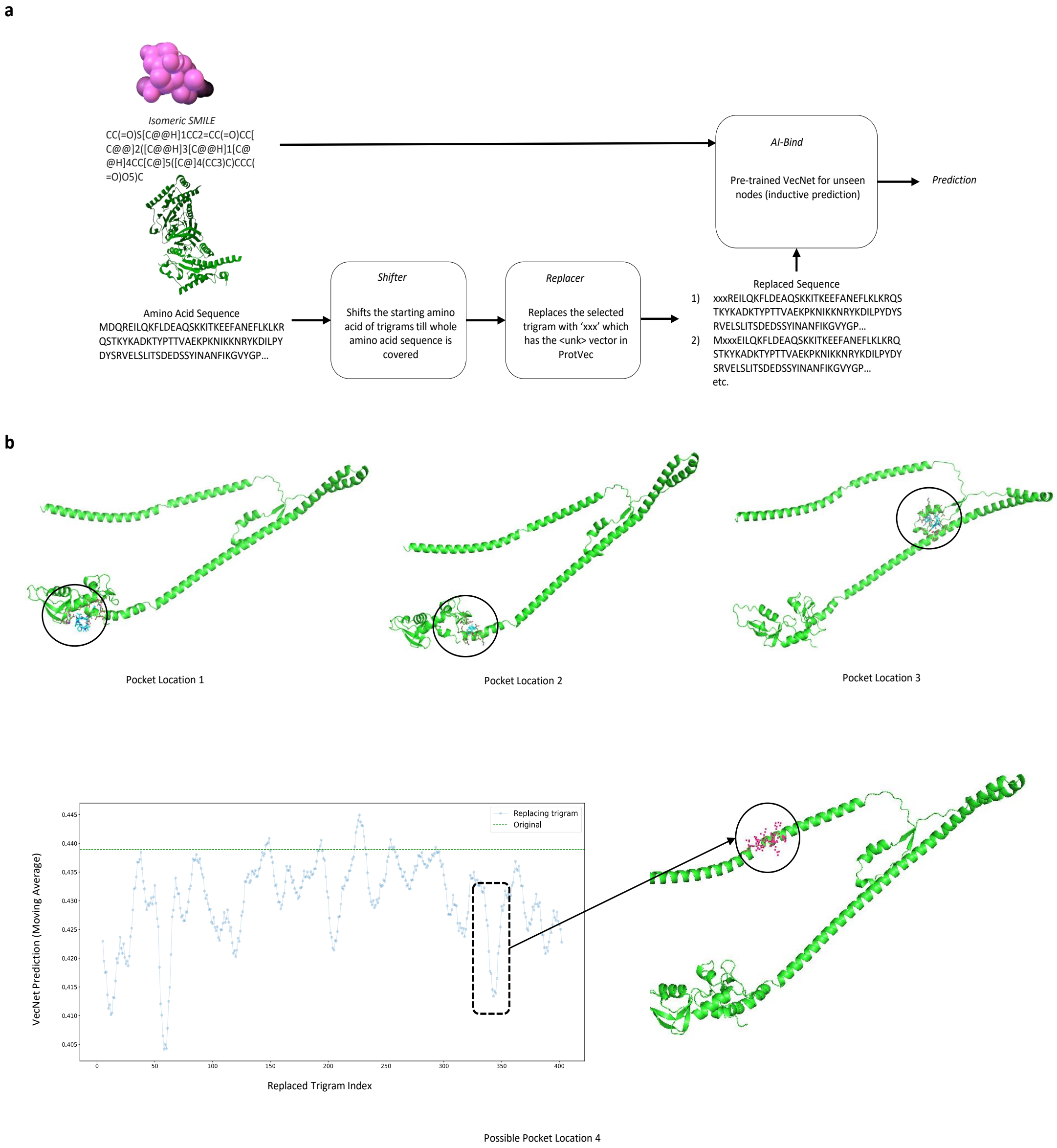

Supplementary Figure 12: **Methodology for interpreting AI-Bind predictions.** **(a)** We replace each amino acid trigram in the amino acid sequence with *xxx*, which maps to the out-of-vocabulary $\langle unk \rangle$ vector in ProtVec, and observe the fluctuations in AI-Bind prediction. Removal of some trigrams affect AI-Bind prediction more than others. These trigrams are indicative of the binding location(s) on the protein. **(b)** We identify three active binding sites on the protein Trim59 from the docking simulations and map them to AI-Bind's binding probability profile. We also identify a possible pocket location from the binding probability profile and visualize that on the 3D protein structure.

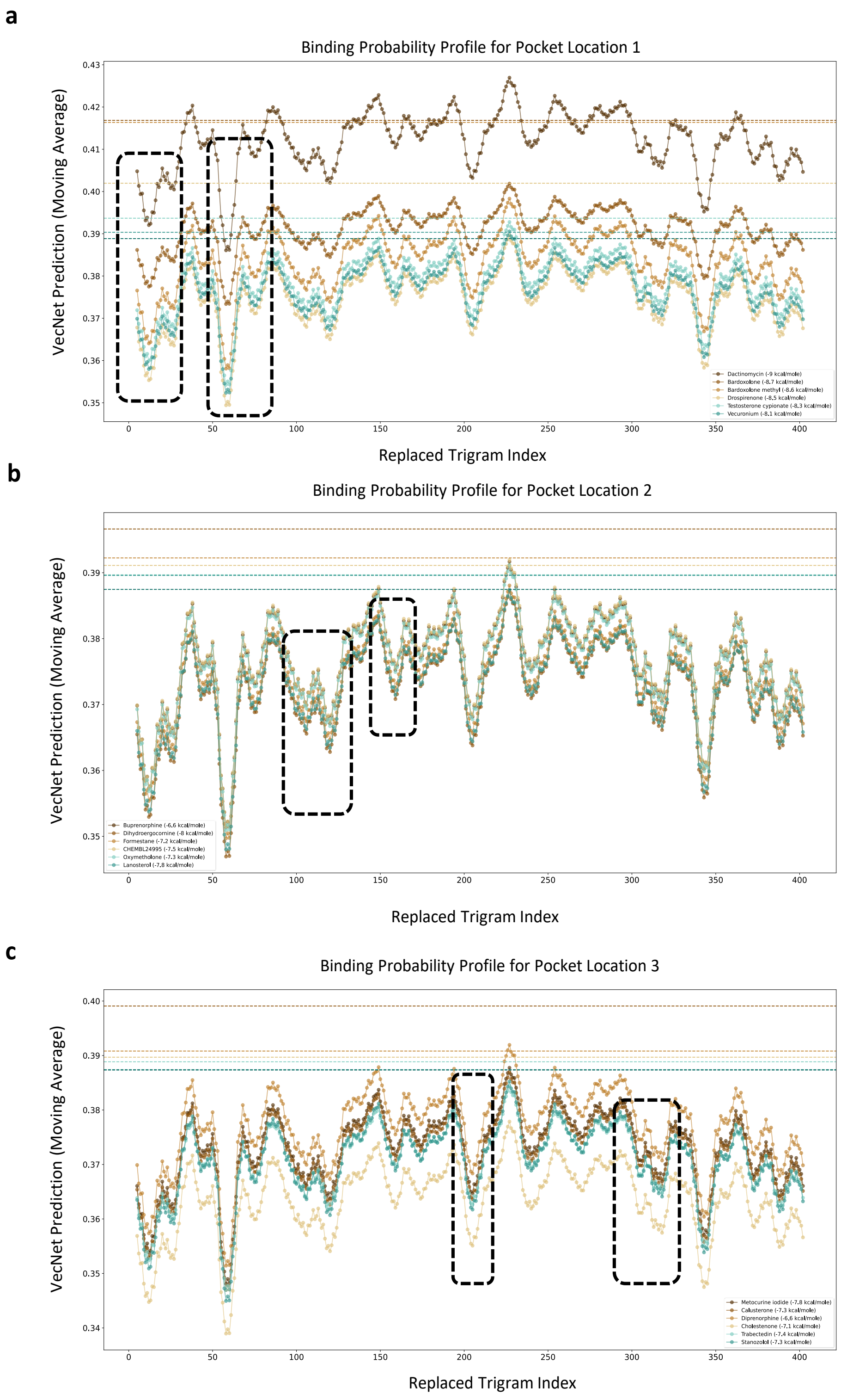

Supplementary Figure 13: **Binding probability profiles for different active binding sites on Trim59. (a)-(c)** We group the ligands based on the binding pockets on Trim59 and plot the binding probability profiles, highlighting the binding locations on the amino acid sequence. We observe a similar shape of the binding probability profiles for different ligands, but the deviation from the original AI-Bind prediction varies across the ligands, which conveys the dependency of the binding probability profile on the ligand structure.

a

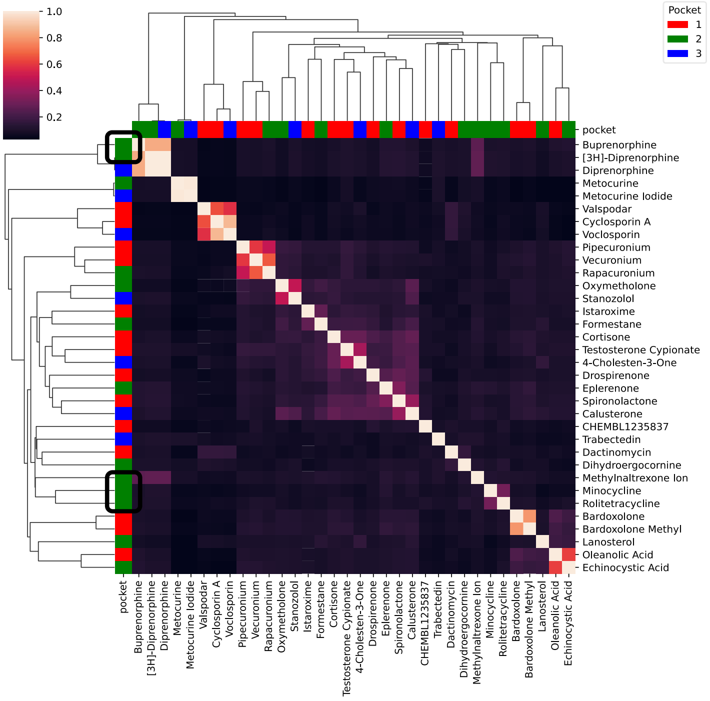

b

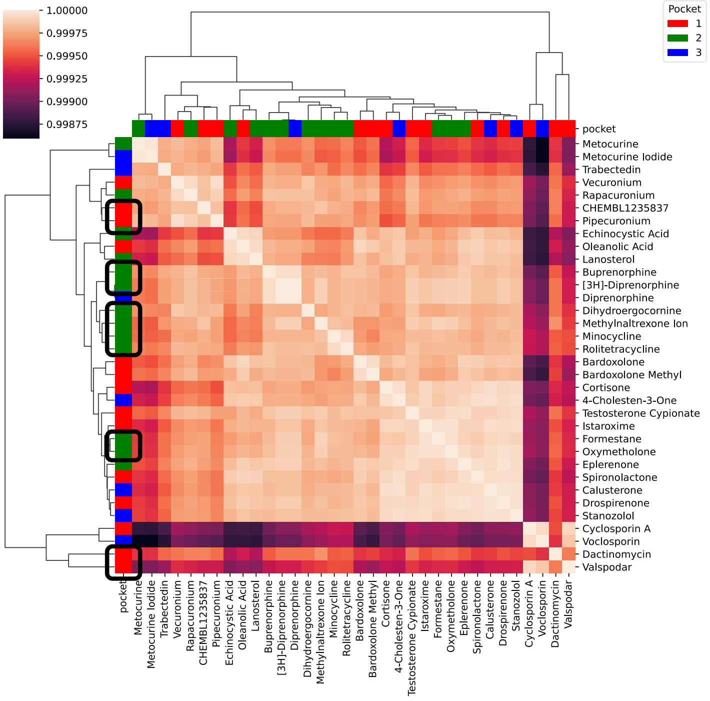

Supplementary Figure 14: **Hierarchical clustering on binding probability profiles. (a)** We plot the heatmap of the Tanimoto similarities between the ligands binding to Trim59. We do not observe a significant grouping of the ligands solely based on their molecular structures. **(b)** We cluster the ligands based on he similarities of their binding probability profiles. We observe that multiple ligands binding to the same pocket are clustered together in the clustermap. Thus, the binding probability profiles generated by AI-Bind are not only specific to a protein, but carry information about the ligand structures.

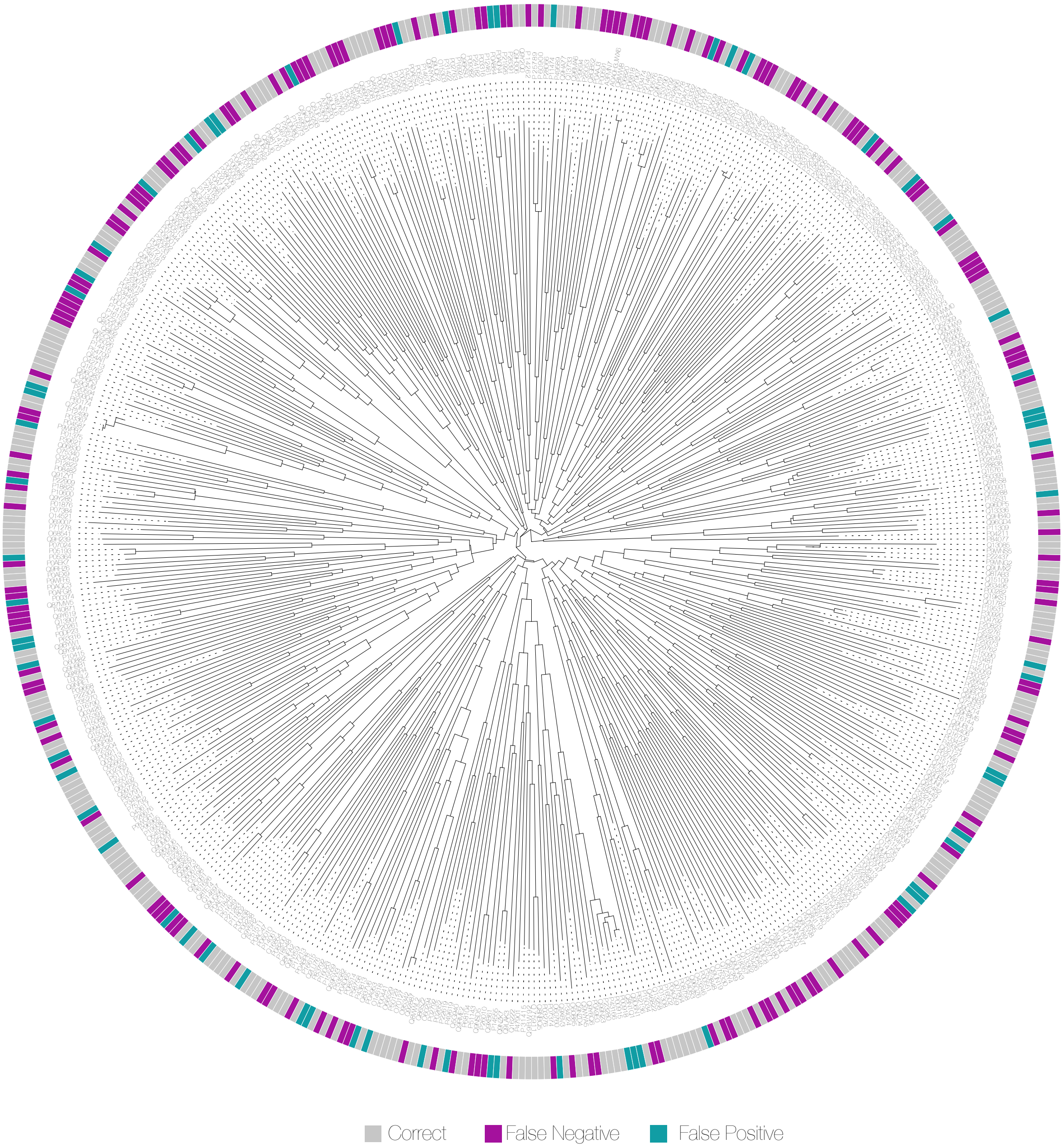

Supplementary Figure 15: **Phylogenetic tree of genes enriched towards prediction bias.** We compare proteins associated with the false predictions (both false positives and false negatives) made by AI-Bind's VecNet to uncover structural similarities. AI-Bind does not show any bias towards certain protein structures in the false predictions, and can be used for binding prediction involving protein structures emerging from different organisms.

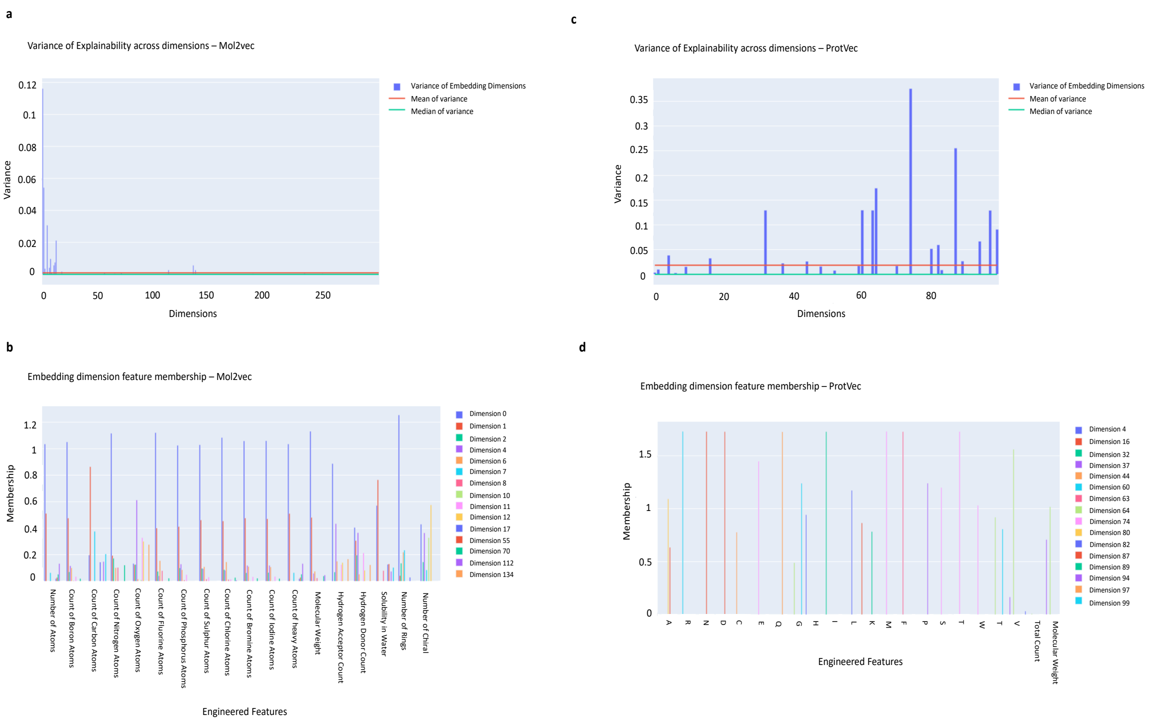

Supplementary Figure 16: **Dimensions of Mol2vec and ProtVec contributing to protein-ligand binding. (a)-(b)** Only 15 Mol2vec dimensions show high variability when explaining the engineered features representing ligand molecules. **(c)-(d)** We find similar results for 16 ProtVec dimensions.

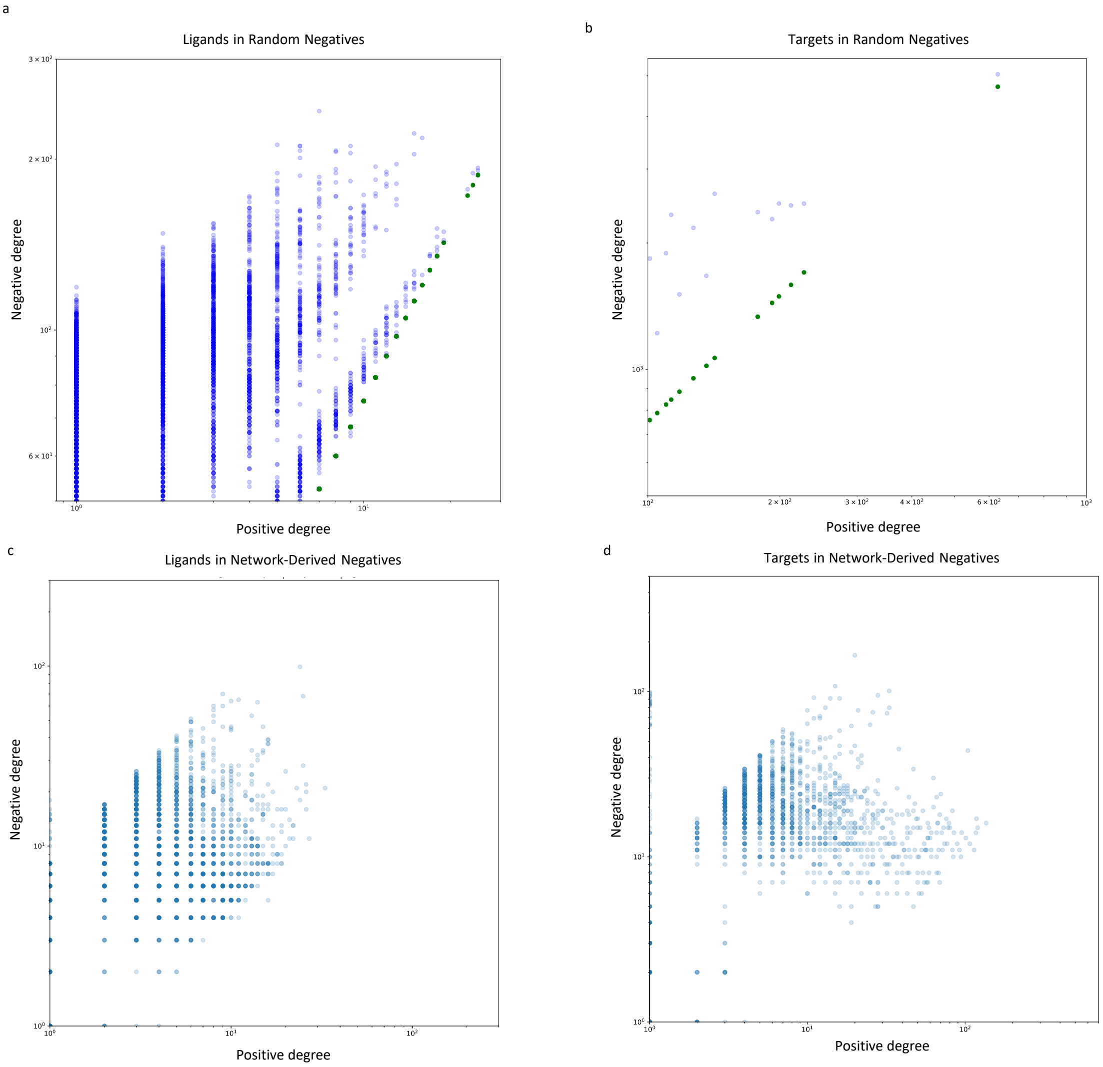

Supplementary Figure 17: **Random vs. network-derived negatives**. **(a)-(b)** In random negative sampling, both the ligand and the protein on a positive edge have the lower bound of negative degree equal to 7.5 times its positive degree. Higher positive degree nodes have lower probabilities of being present in a random negative sample, as they are present in many positive edges and are discarded more often from getting included in a negative sample. Thus, the negative degree diminishes as the positive degree increases. **(c)-(d)** We observe less correlation between positive and negative degrees for the network-derived negatives. This helps in removing the annotation imbalance we observe in the existing protein-ligand databases.

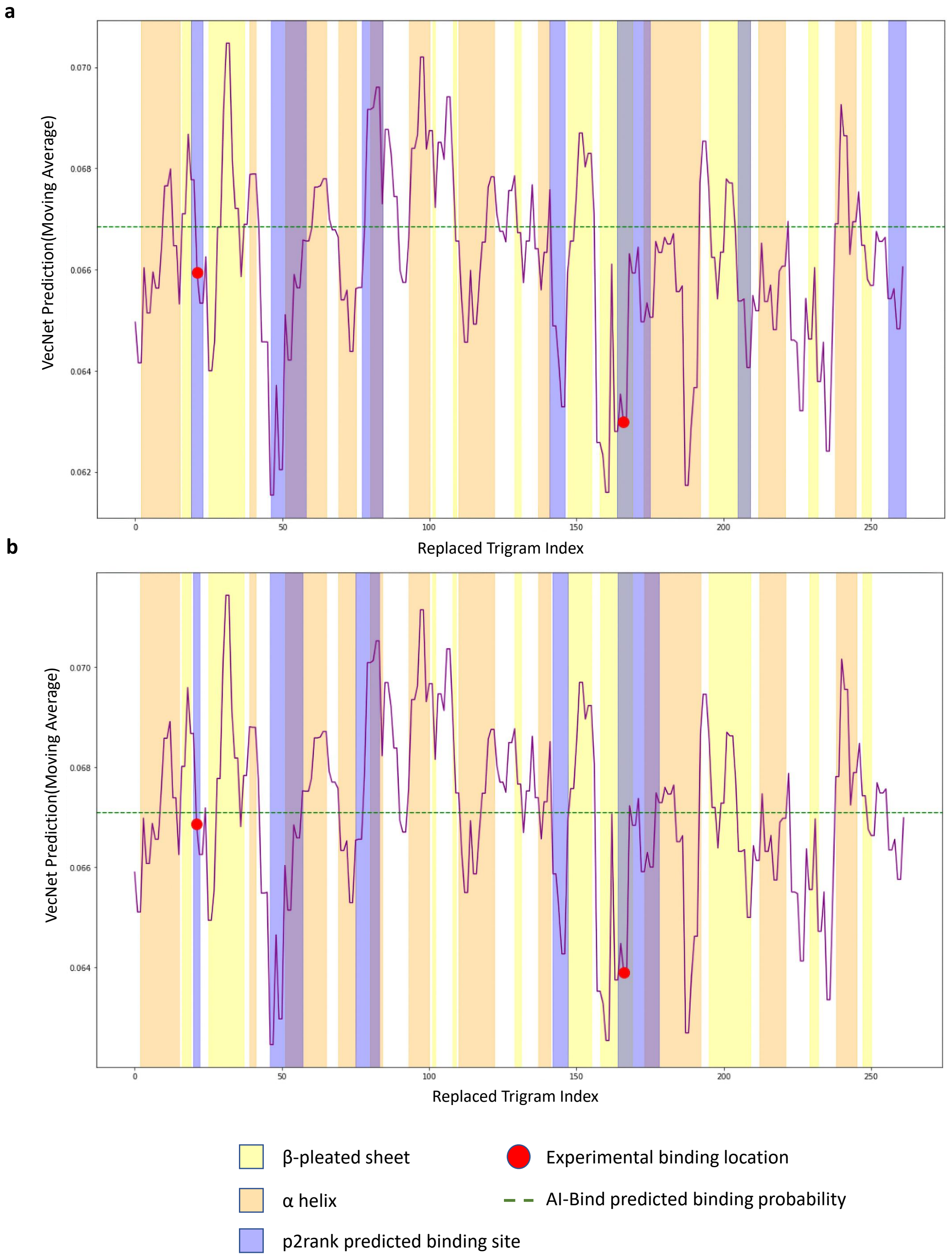

Supplementary Figure 18: **Experimental binding sites lie on the valleys of the binding probability profile.** We plot the binding probability profile for the *E. Coli* protein Thymidylate Synthase, and the ligands **(a)** SP-722 and **(b)** SP-876. We observe that the experimentally obtained binding sites are in the valleys of the binding probability profile, and overlay on the β-sheets and the coils regions. These binding locations also overlap with the binding locations predicted by P2Rank, a state-of-the-art binding site prediction algorithm.

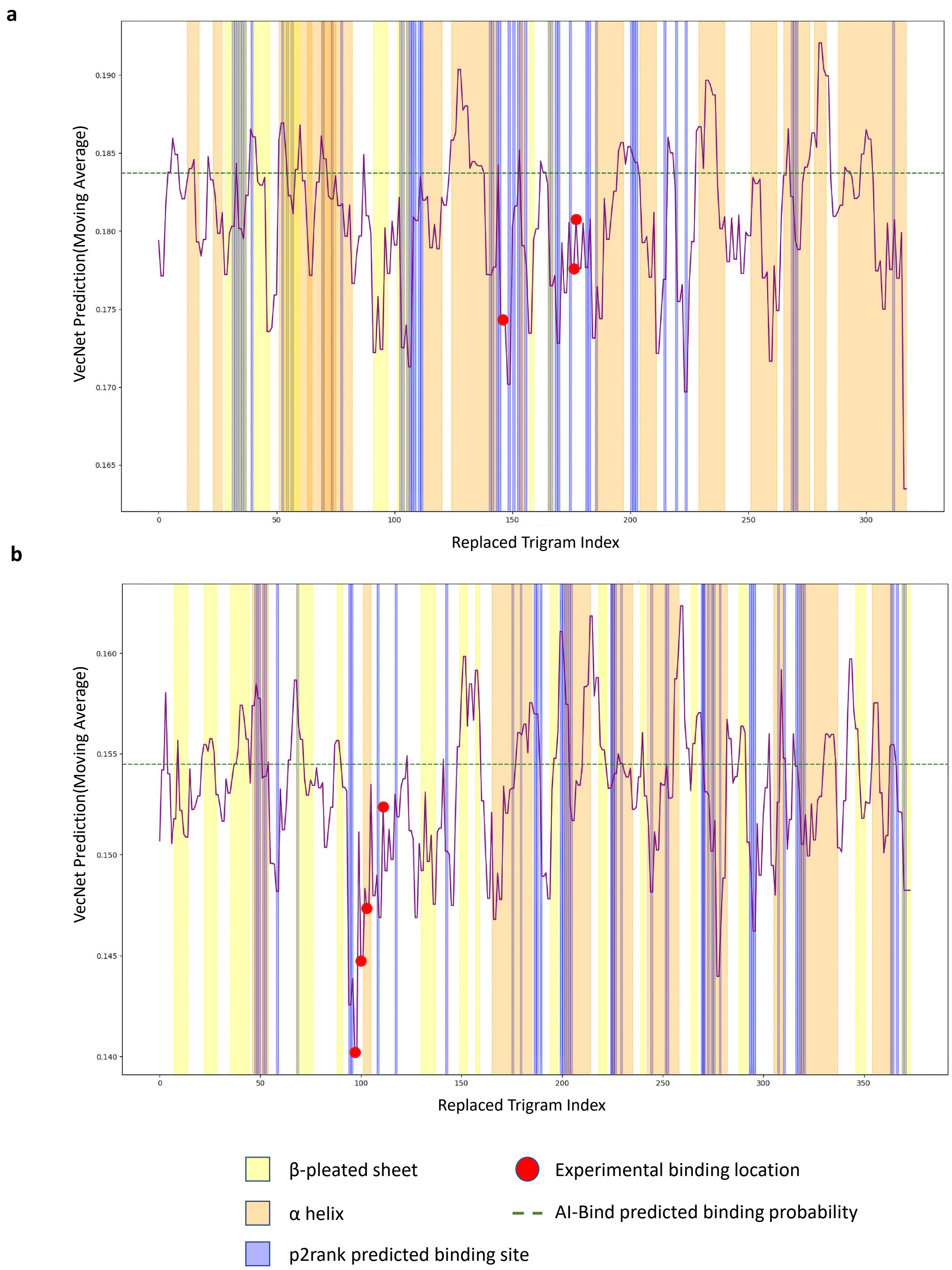

Supplementary Figure 19: **Experimental binding sites lie on the valleys of the binding probability profile for human proteins TAO3 Kinase and Human Alcohol Dehydrogenase.** We plot the binding probability profile for the human protein and ligand pairs **(a)** Human TAO3 Kinase and ADP **(b)** Human Alcohol Dehydrogenase and Nicotinamide Adenine Dinucleotide. We observe that the experimentally obtained binding sites are in the valleys of the binding probability profile, and often overlay on the β-sheets and the coils regions. These binding locations also overlap with the binding locations predicted by P2Rank, a state-of-the-art binding site prediction algorithm.
