# Supplementary material for: Improving the generalizability of protein-ligand binding predictions with AI-Bind
Source: Nat Commun. 2023 Apr 8;14:1989. doi: 10.1038/s41467-023-37572-z (PMC10082765; doi:10.1038/s41467-023-37572-z)
Supplement: Supplementary file 3 — Description of Additional Supplementary Files [file 41467_2023_37572_MOESM3_ESM.docx]

**Supplementary Dataset 1**

This file contains the docking validation of the top 100 protein-ligand pairs predicted by AI-Bind’s VecNet. Using the 3D structures of the viral and the human proteins associated with SARS-CoV-2 found in PDB and UniProt, we derive the binding affinities in kcal mol^-1^ for 74 pairs in the top predictions. We report the gene names, the ligand names, and the corresponding binding affinities in this file.

**Supplementary Dataset 2**

This file contains the list of the top 100 protein-ligand pairs predicted by AI-Bind’s VecNet on the viral and the human proteins associated with SARS-CoV-2. We report the InChIKeys of the ligands, the amino acid sequences of the proteins, and the corresponding gene names.

**Supplementary Dataset 3**

This file contains the list of the bottom 100 protein-ligand pairs predicted by AI-Bind’s VecNet on the viral and the human proteins associated with SARS-CoV-2. We report the InChIKeys of the ligands, the amino acid sequences of the proteins, and the corresponding gene names.
