# Supplementary material for: Improving the generalizability of protein-ligand binding predictions with AI-Bind
Source: Nat Commun. 2023 Apr 8;14:1989. doi: 10.1038/s41467-023-37572-z (PMC10082765; doi:10.1038/s41467-023-37572-z)
Supplement: Supplementary file 7 — Reporting Summary [file 41467_2023_37572_MOESM7_ESM.pdf]

nature portfolio

Corresponding author(s): Giulia Menichetti

Last updated by author(s): Jan 25, 2023

Reporting Summary

Nature Portfolio wishes to improve the reproducibility of the work that we publish. This form provides structure for consistency and transparency in reporting. For further information on Nature Portfolio policies, see our Editorial Policies and the Editorial Policy Checklist.

Statistics

For all statistical analyses, confirm that the following items are present in the figure legend, table legend, main text, or Methods section.

n/a	Confirmed	
☐	☒	The exact sample size (*n*) for each experimental group/condition, given as a discrete number and unit of measurement
☐	☒	A statement on whether measurements were taken from distinct samples or whether the same sample was measured repeatedly
☐	☒	The statistical test(s) used AND whether they are one- or two-sided<br>*Only common tests should be described solely by name; describe more complex techniques in the Methods section.*
☐	☒	A description of all covariates tested
☐	☒	A description of any assumptions or corrections, such as tests of normality and adjustment for multiple comparisons
☐	☒	A full description of the statistical parameters including central tendency (e.g. means) or other basic estimates (e.g. regression coefficient) AND variation (e.g. standard deviation) or associated estimates of uncertainty (e.g. confidence intervals)
☐	☒	For null hypothesis testing, the test statistic (e.g. *F*, *t*, *r*) with confidence intervals, effect sizes, degrees of freedom and *P* value noted<br>*Give P values as exact values whenever suitable.*
☒	☐	For Bayesian analysis, information on the choice of priors and Markov chain Monte Carlo settings
☒	☐	For hierarchical and complex designs, identification of the appropriate level for tests and full reporting of outcomes
☒	☐	Estimates of effect sizes (e.g. Cohen's *d*, Pearson's *r*), indicating how they were calculated

Our web collection on statistics for biologists contains articles on many of the points above.

Software and code

Policy information about availability of computer code

Data collection

The first part of the paper uses data from DeepPurpose and MolTrans, which is derived from BindingDB. The data is downloadable from the DeepPurpose paper [Huang, K. et al. DeepPurpose: a deep learning library for drug–target interaction prediction. Bioinformatics 36, 5545–5547 (2020)]. AI-Bind uses multiple databases (DrugBank, BindingDB, Drug Target Commons) as training data. The data is readily downloadable from their websites: DrugBank (https://www.drugbank.com/), BindingDB (https://www.bindingdb.org), Drug Target Commons (http://drugtargetcommons.fimm.fi/). We obtain the amino acid sequences and 3D proteins structures from Uniprot (https://www.uniprot.org/) and Protein Data Bank (https://www.rcsb.org/). We use PubChem (https://pubchem.ncbi.nlm.nih.gov/) for the collecting of ligand information. The published papers for each of these databases are cited in our References List. We use the amino acid sequences and InChIKeys as identifiers for proteins and compounds respectively, allowing us to match entries between the different databases. No specialized software was used to collect the data.

Data analysis

All the code for using AI-Bind to make protein-ligand binding predictions and reproducing the results is available in our GitHub and syned with Zenodo at: https://doi.org/10.5281/zenodo.7563566.500. The data analysis is performed using Python 3.6.6 with CUDA v9.0 and rdkit 2017.09.1 except for the duplex configuration model which was performed using MATLAB R2021a. For the docking simulations we used AutoDock Vina 1.1.2 and AutoDockTools-1.5.7. AI-Bind is developed upon a collection of python packages which can be found in the GitHub and as list below:
absl-py == 0.7.1
asn1crypto == 0.24.0
astor == 0.7.1
attrs == 19.3.0
autograd == 1.3
autograd-gamma == 0.4.2

backcall == 0.1.0
bazel == 0.0.0.20200723
beautifulsoup4 == 4.9.1
bicm == 2.0.3
biopython == 1.71
biothings-client == 0.2.1
bleach == 3.1.4
blessings == 1.7
bokeh == 2.0.2
boto == 2.49.0
boto3 == 1.14.12
botocore == 1.17.12
bs4 == 0.0.1
category-encoders
certifi == 2018.8.24
cffi == 1.12.3
chardet == 3.0.4
chembl-webresource-client == 0.10.1
chromedriver-binary == 77.0.3865.40.0
chromedriver-binary-auto == 0.1.2
click == 8.0.4
cloudpickle == 1.3.0
copulalib == 1.1.0
copulas
cryptography == 2.5
cspy == 0.0.14
cycler == 0.10.0
dask == 2.14.0
dataclasses == 0.8
deap == 1.3.1
decorator == 4.4.0
deepchem == 2.4.0.dev20201222014716
defusedxml == 0.6.0
dill == 0.3.1.1
distributed == 2.14.0
docutils == 0.15.2
easydict == 1.9
EasyProcess == 0.3
ensembl-rest == 0.3.3
entrypoint2 == 0.2.1
entrypoints == 0.3
fa2 == 0.3.5
flaky == 3.3.0
Flask == 1.0.2
fsspec == 0.7.3
future == 0.18.2
gast == 0.2.2
gensim == 3.8.3
glove-python == 0.1.0
gpustat == 0.6.0
graphviz == 0.13.2
grpcio == 1.20.1
gunicorn == 19.9.0
h5py == 2.9.0
HeapDict == 1.0.1
idna == 2.6
imbalanced-learn == 0.6.2
imblearn == 0.0
importlib-metadata == 1.5.0
importlib-resources == 1.4.0
intervaltree == 3.0.2
ipykernel == 5.1.4
ipython == 7.13.0
ipython-genutils == 0.2.0
ipywidgets == 7.5.1
itsdangerous == 2.0.1
jedi == 0.16.0
Jinja2 == 3.0.3
jmespath == 0.10.0
joblib == 0.11
jsonschema == 3.2.0
jupyter == 1.0.0
jupyter-client == 6.1.2
jupyter-console == 6.1.0
jupyter-contrib-core == 0.3.3
jupyter-contrib-nbextensions == 0.5.1

```
jupyter-core == 4.6.3
jupyter-highlight-selected-word == 0.2.0
jupyter-latex-envs == 1.4.6
jupyter-nbextensions-configurator == 0.4.1
Keras == 2.3.1
Keras-Applications == 1.0.7
Keras-Preprocessing == 1.0.9
kiwisolver == 1.1.0
lifelines == 0.24.6
littleutils == 0.2.2
llvmlite == 0.36.0
locket == 0.2.0
lxml == 4.5.0
Markdown == 3.1
MarkupSafe == 2.0.1
matlab == 0.1
matplotlib == 3.2.1
matplotlib-venn == 0.11.5
mistune == 0.8.4
mkl-fft == 1.0.6
mkl-random == 1.0.1
git+https://github.com/samoturk/mol2vec
mordred == 1.2.0
mpmath == 1.2.1
msgpack == 1.0.0
mygene == 3.1.0
nbconvert == 5.6.1
nbformat == 5.0.4
networkx == 2.1
nltk == 3.6.5
nose == 1.3.7
nose-timer == 0.7.0
notebook == 6.0.3
numba == 0.53.1
numexpr == 2.6.9
numpy == 1.18.3
nvidia-ml-py3 == 7.352.0
olefile == 0.46
ordered-set == 4.0.2
outdated == 0.2.1
packaging == 21.3
pandarallel == 1.4.8
pandas == 1.1.5
pandocfilters == 1.4.2
parso == 0.6.2
partd == 1.1.0
patool == 1.12
patsy == 0.5.1
pbr == 3.1.1
pexpect == 4.8.0
pickleshare == 0.7.5
Pillow == 5.0.0
pip == 19.1
plotly == 4.14.3
plotly-express == 0.4.1
powerlaw == 1.5
prometheus-client == 0.7.1
prompt-toolkit == 3.0.4
protobuf == 3.7.1
psutil == 5.7.0
ptyprocess == 0.6.0
PubChemPy == 1.0.4
py7zr == 0.9.5
pyaml == 21.10.1
pycosat == 0.6.3
pycparser == 2.19
pycryptodome == 3.9.8
pydot == 1.4.1
Pygments == 2.6.1
pynndescent == 0.5.4
pyOpenSSL == 19.0.0
pyparsing == 2.4.0
pyrsistent == 0.16.0
PySocks == 1.6.8
python-dateutil == 2.8.0
pytz == 2019.1
```

pyunpack == 0.2.1
PyYAML == 5.3.1
pyzmq == 18.1.1
qtconsole == 4.7.2
QtPy == 1.9.0
regex == 2021.11.10
requests == 2.25.1
requests-cache == 0.5.2
retrying == 1.3.3
ruamel-yaml == 0.15.46
s3transfer == 0.3.3
scikit-learn == 0.23.2
scikit-optimize == 0.9.0
scipy == 1.4.1
seaborn == 0.10.0
selenium == 3.141.0
Send2Trash == 1.5.0
setuptools == 39.0.1
shap == 0.40.0
simdna == 0.4.2
simplejson == 3.17.0
six == 1.16.0
sklearn == 0.0
slicer == 0.0.7
smart-open == 2.0.0
sortedcontainers == 2.1.0
soupsieve == 2.0.1
statsmodels == 0.12.2
subword-nmt == 0.3.7
swifter == 0.302
tables == 3.5.1
TBB == 2021.6.0
tblib == 1.6.0
tensorboard == 1.12.2
tensorflow-gpu == 1.12.0
termcolor == 1.1.0
terminado == 0.8.3
testpath == 0.4.4
texttable == 1.6.2
threadpoolctl == 2.0.0
toolz == 0.10.0
torch == 1.8.1
tornado == 6.0.2
tqdm == 4.60.0
traitlets == 4.3.3
typing-extensions == 3.7.4.2
umap == 0.1.1
umap-learn == 0.5.1
update-checker == 0.18.0
urllib3
venn == 0.1.3
wcwidth == 0.1.9
webencodings == 0.5.1
Werkzeug == 2.0.3
wget == 3.2
wheel == 0.33.1
widgetsnbextension == 3.5.1
xgboost == 0.6a2
xlrd == 2.0.1
zict == 2.0.0
zipp == 2.2.0

For manuscripts utilizing custom algorithms or software that are central to the research but not yet described in published literature, software must be made available to editors and reviewers. We strongly encourage code deposition in a community repository (e.g. GitHub). See the Nature Portfolio guidelines for submitting code & software for further information.

Data

Policy information about availability of data

All manuscripts must include a data availability statement. This statement should provide the following information, where applicable:

- Accession codes, unique identifiers, or web links for publicly available datasets
- A description of any restrictions on data availability
- For clinical datasets or third party data, please ensure that the statement adheres to our policy

The data generated and analyzed in the study have been deposited on Zenodo at https://zenodo.org/record/7226641. The top 100 and bottom 100 binding

predictions from AI-Bind on the COVID-19 related proteins are available within the Supplementary Files. A Source Data File is provided with this manuscript. The publicly available datasets used in this study can be found on their associated websites: DrugBank (https://www.drugbank.com/), BindingDB (https://www.bindingdb.org), Drug Target Commons (http://drugtargetcommons.fimm.fi/), Uniprot (https://www.uniprot.org/), Protein Data Bank (https://www.rcsb.org/), and PubChem (https://pubchem.ncbi.nlm.nih.gov/).

Human research participants

Policy information about studies involving human research participants and Sex and Gender in Research.

Reporting on sex and gender	NA
Population characteristics	NA
Recruitment	NA
Ethics oversight	NA

Note that full information on the approval of the study protocol must also be provided in the manuscript.

Field-specific reporting

Please select the one below that is the best fit for your research. If you are not sure, read the appropriate sections before making your selection.

☒ Life sciences ☐ Behavioural & social sciences ☐ Ecological, evolutionary & environmental sciences

For a reference copy of the document with all sections, see nature.com/documents/nr-reporting-summary-flat.pdf

Life sciences study design

All studies must disclose on these points even when the disclosure is negative.

Sample size	BindingDB data has 10,000 ligands and 1,400 proteins. We combined multiple open-source and publicly accessible databases (DrugBank + BindingDB + Drug Target Commons) to create the AI-Bind training data, which consists of 8,000 ligands and 5,000 proteins. These are widely cited and used as benchmark datasets for protein-ligand binding assessment tasks. This data is sufficient enough for the purposes of the paper because they provide us with enough data points for train-test-validation split and significant number of tests samples to compute cross-validation performance.
Data exclusions	We use InChIKeys and amino acid sequences as the unique identifiers for ligands and proteins respectively. Positive and negative samples are selected from DrugBank, BindingDB, and Drug Target Commons (DTC). We consider samples from Binding DB and DTC to be binding or non-binding based on the kinetic constants Ki, Kd, IC50, and EC50. We use thresholds of ≤ 10^3 nM and ≥ 10^6 nM to obtain positive and (absolute) negative annotations. We then filter out all samples outside of the temperature range 20∘C - 45∘C to remove protein-ligand pairs that likely use denatured proteins (i.e. unraveled proteins at high temperatures and restructured proteins at cold temperatures). This is done to ensure the proteins are in their functional structure as found within the body.
Replication	DeepPurpose, MolTrans, and AI-Bind performances were all assessed using 5-fold cross validation, each fold containing its own unique set of protein-ligand pairs. In this way each fold is a replication of the performances for each machine learning model. The results of each fold are shown as dot plots in the figures.<br><br>Next, we validate the AI-Bind pipeline by predicting potential ligands binding to SARS-CoV-2 viral proteins and human proteins they are known to bind with. The AI-Bind predictions are then validated via docking simulations. The top and bottom predictions (total of 128 docking simulations) with available protein and ligand structures were validated. We ran AI-Bind on 26 SARS-CoV-2 viral proteins and the 332 human proteins targeted by the SARS-CoV-2 viral proteins over 8,000 ligands, and then validated the top and bottom 100 predictions.
Randomization	For training and testing of all the machine learning models used in the paper, we created a random split of 85:15 over 5-folds, where 85% of the data is used in training and the remaining 15% is used in validation and testing.
Blinding	In the building of the machine learning models, they are trained on 85% of the data and then their performance is tested on the remaining 15% of the data (i.e. blind to 15% of the protein-ligand interactions within the dataset). In addition, we do blinding studies in the form of inductive link prediction by testing the performance of the models on proteins and ligands unseen during the training phase. Lastly, we further validate AI-Bind on SARS-CoV-2 proteins, which the model did not see during its training.

Reporting for specific materials, systems and methods

We require information from authors about some types of materials, experimental systems and methods used in many studies. Here, indicate whether each material, system or method listed is relevant to your study. If you are not sure if a list item applies to your research, read the appropriate section before selecting a response.

Materials & experimental systems

n/a	Involved in the study
☒	☐ Antibodies
☒	☐ Eukaryotic cell lines
☒	☐ Palaeontology and archaeology
☒	☐ Animals and other organisms
☒	☐ Clinical data
☒	☐ Dual use research of concern

Methods

n/a	Involved in the study
☒	☐ ChIP-seq
☒	☐ Flow cytometry
☒	☐ MRI-based neuroimaging
